# Opposing USP19 splice variants in TGF-β signaling and TGF-β-induced epithelial–mesenchymal transition of breast cancer cells

**DOI:** 10.1007/s00018-022-04672-w

**Published:** 2023-01-17

**Authors:** Jing Zhang, Maarten van Dinther, Midory Thorikay, Babak Mousavi Gourabi, Boudewijn P. T. Kruithof, Peter ten Dijke

**Affiliations:** 1 Oncode Institute, Leiden University Medical Center, 2300 RC Leiden, The Netherlands; 2 Department of Cell Chemical Biology, Leiden University Medical Center, 2300 RC Leiden, The Netherlands; 3 Department of Anatomy and Embryology, Leiden University Medical Center, 2300 RC Leiden, The Netherlands; 4 HARTZ, Leiden University Medical Center, 2300 RC Leiden, The Netherlands

**Keywords:** USP19, Alternative spliced isoform, Transforming growth factor-β, Epithelial–mesenchymal transition, Breast cancer, Herboxidiene

## Abstract

Ubiquitin-specific protease (USP)19 is a deubiquitinating enzyme that regulates the stability and function of multiple proteins, thereby controlling various biological responses. The alternative splicing of USP19 results in the expression of two major encoded variants that are localized to the endoplasmic reticulum (ER) (USP19-ER) and cytoplasm (USP19-CY). The importance of alternative splicing for the function of USP19 remains unclear. Here, we demonstrated that USP19-CY promotes TGF-β signaling by directly interacting with TGF-β type I receptor (TβRI) and protecting it from degradation at the plasma membrane. In contrast, USP19-ER binds to and sequesters TβRI in the ER. By decreasing cell surface TβRI levels, USP19-ER inhibits TGF-β/SMAD signaling in a deubiquitination-independent manner. Moreover, USP19-ER inhibits TGF-β-induced epithelial–mesenchymal transition (EMT), whereas USP19-CY enhances EMT, as well as the migration and extravasation of breast cancer cells. Furthermore, USP19-CY expression is correlated with poor prognosis and is higher in breast cancer tissues than in adjacent normal tissues. Notably, the splicing modulator herboxidiene inhibits USP19-CY, increases USP19-ER expression and suppresses breast cancer cell migration. Targeting USP19 splicing or its deubiquitinating activity may have potential therapeutic effects on breast cancer.

**Supplementary Information:**

The online version contains supplementary material available at 10.1007/s00018-022-04672-w.

## Introduction

Transforming growth factor-β (TGF-β) is a multifunctional cytokine that plays essential roles in the development and homeostasis of most human tissues [1, 2]. Disruption of TGF-β signaling has been linked to a multitude of human diseases, including cancer [2, 3]. TGF-β signaling is initiated by the binding of TGF-β to the extracellular domain of the transmembrane TGF-β type II receptor (TβRII), which has serine/threonine kinase activity [4, 5]. Then, TGF-β type I receptor (TβRI) is recruited and forms a heteromeric complex with TβRII, which then is transphosphorylated by the TβRII kinase [6]. Activation of the TβRII/TβRI complex phosphorylates Sma- and Mad-related (SMAD) proteins, i.e., SMAD2 and SMAD3, at two carboxy-terminal serine residues. These phosphorylated R-SMADs form complexes with a common SMAD mediator, namely, SMAD4, and translocate to the nucleus to interact with high-affinity DNA-binding transcription factors and chromatin remodeling proteins, thereby modulating the transcription of TGF-β target genes, including *SMAD7*, *SERPINE1* and *CCN2*, that encode the SMAD7, PAI1, and CTGF proteins, respectively [7, 8]. TGF-β is a strong driver of epithelial–mesenchymal transition (EMT), which is a dynamic and reversible process in which epithelial cells lose their cell‒cell contacts and apical-basal polarity and acquire mesenchymal phenotypes with enhanced migratory abilities [9]. EMT is characterized by the downregulation of epithelial markers, i.e., E-cadherin and claudin-1, and the upregulation of mesenchymal markers, i.e., *N*-cadherin, vimentin, and SNAIL1/2 [10]. Incomplete EMT is referred to as epithelial–mesenchymal plasticity (EMP) [11]. EMT plays a critical role in embryonic development [12] and cancer cell migration, invasion and metastasis [13–15].

The ubiquitination of TGF-β signaling pathway components, including its receptors, is a posttranslational modification that is emerging as a key mechanism by which TGF-β signaling is regulated [16, 17]. Ubiquitination depends on a cascade of enzymes that includes ubiquitin-activating enzymes (E1), ubiquitin-conjugating enzymes (E2), and ubiquitin ligases (E3), which mediate the transfer of ubiquitin to protein substrates [18]. The TGF-β target gene *SMAD7*, which is a negative regulator of TGF-β signaling, binds to the SMURF E3 ubiquitin ligases and recruits it to TβRI; this leads to the SMURF-mediated polyubiquitylation of the receptor for degradative endocytosis [19–21]. Deubiquitinases (DUBs) directly oppose the conjugating function of E3 ligases by removing ubiquitin chains from target proteins [22]. The DUBs ubiquitin-specific protease (USP)4 and USP15 have been shown to antagonize the SMAD7/SMURF2-mediated polyubiquitination and proteasomal degradation of TβRI. Although USP4 was found to directly interact with TβRI [23], USP15 is recruited to TβRI by SMAD7 [24, 25]. In addition, USP4 was found to interact with USP11, USP15, and USP19, and these DUBs cooperate in the deubiquitination of polyubiquitinated TβRI [23].

USP19 is unique among the DUB family, which has over 100 members, in that it contains a carboxy-terminal transmembrane (TM) domain that targets USP19 to the endoplasmic reticulum (ER), where its active site face the cytosol [26]. In addition to this USP19-ER isoform, USP19 is expressed as another major isoform that lacks the TM domain; this isoform localizes to the cytoplasm (herein referred to as USP19-CY) [27]. The USP19-ER and USP19-CY isoforms are produced via alternative splicing of the last exon of the *USP19* gene [28]. Structurally, both isoforms possess two CHORD-SGT1 (CS)/P23 domains in their N-termini that serve as cochaperones of Hsp90 [29]; a central USP domain with deubiquitinating activity that harbors the essential cysteine (C), aspartic acid (D), histidine (H) amino acid residues; a putative ubiquitin-like (UBL) domain; and a MYND Zn finger domain [30–32]. Multiple studies about USP19 have focused on the USP19-ER variant and its role in the unfolded protein response, which prevents the ER-associated degradation (ERAD) of substrates [27]. In addition, USP19 deubiquitinates and thereby regulates the stability of the ubiquitin ligase KPC1, the inhibitors of apoptosis c-IAP1 and c-IAP2, and hypoxia inducible factor 1α (HIF-1α) under hypoxic conditions [33–35]. However, whether the differential localization of USP19 impacts TGF-β signaling and its role in TGF-β-induced EMT, cell migration and invasion of cancer cells remain unclear. Therefore, in this study, we investigated the roles of these two USP19 splicing variants and demonstrated their opposing actions in TGF-β-induced responses. We also elucidated the underlying mechanism by which the USP19-CY isoform promotes TGF-β signaling by deubiquitinating and stabilizing TβRI, thereby enhancing EMT, cell migration and invasion. However, USP19-ER inhibits TGF-β-induced responses in a DUB activity-independent manner by sequestering TβRI in the ER.

## Materials and methods

### Cell culture

HEK 293T cells, human A549-VIM-RFP lung adenocarcinoma cells, MDA-MB-231 breast cancer cells and human U2OS osteosarcoma cells were originally obtained from American Type Culture Collection (ATCC) and cultured in Dulbecco’s modified Eagle medium (DMEM, 11965092, Thermo) supplemented with 10% fetal bovine serum (FBS, S1860-500, BioWest) and 100 U/mL penicillin‒streptomycin (15140148, Thermo). The MCF10A-Ras breast epithelial cell line was derived from MCF10A cells that were transformed with Ha-Ras (kindly provided by Dr. Fred Miller) (Barbara Ann Karmanos Cancer Institute, Detroit, MI), and cultured in DMEM/F12 (11039047, Thermo) supplemented with l-glutamine with 5% horse serum (26050088, Thermo), 20 ng/mL epidermal growth factor (EGF 01–107, Merck Millipore), 10 mg/mL insulin (91077C, Sigma), 100 ng/mL cholera enterotoxin (C8052, Sigma), 0.5 mg/mL hydrocortisone (H0135, Sigma), and 100 U/mL penicillin‒streptomycin. All the cell lines were tested to confirm the absence of mycoplasma contamination and were authenticated by short tandem repeat (STR) profiling.

### Reagents and antibodies

The splicing modulators that were used were SRPIN340 (5042930001, Sigma), TG003 (T5575, Sigma), indisulam (SML1225, Sigma), GSK3326595 (GSK, HY-101563, MedChemExpress), T025 (HY-112296, MedChemExpress), URMC-099 (HY-12599, MedChemExpress), herboxidiene (10-1614, Focus Biomolecules) and Sudemycin D6 (provided by Dr. A.G. Jochemsen, LUMC). Cycloheximide (CHX) was obtained from Sigma (66-81-9). TGF-β3 was generously provided by Dr. A. Hinck (University of Pittsburgh, PA). Biotin (21335) was obtained from Thermo. The antibodies used for immunoprecipitation (IP), immunoblotting (IB), and immunofluorescence (IF) were as follows: phosphor-SMAD2 (1:1000; IB; 3108, Cell Signaling), total-SMAD2 (1:1000; IB; 3103S, Cell Signaling), USP19 (1:1000; IB; IF: ab189518, Abcam), GAPDH (1:1000; IB; MAB374, Millipore), Tubulin (1:1000; IB; 2148, Cell Signaling), E-cadherin (1:1000; IB; 610181, BD Biosciences), N-cadherin (1:1000; IB; 610920, BD Biosciences), vimentin (1:1000; IB; 5741, Cell Signaling), SNAIL (1:1000; IB; 3879, Cell Signaling), vinculin (1:1000; IB; V9131, Sigma), c-MYC (1:200; IP; sc-40, Santa Cruz), FLAG (1:1000; IB; F3165), HA (1:1000; IB; 1583816, Roche), TβRI (1:1000; IB; sc-398, Santa Cruz), calnexin (1:1000; IF; ab22595, Abcam), Alexa Fluor 555 secondary antibody (1:250 or 1:1000; IF; A-31572, Thermo), Alexa Fluor 488 secondary antibody (1:1000; IF; A-11001, Thermo).

The antibodies against USP19-CY and USP19-ER were raised in rabbits and purified by Eurogentec. The following USP19-CY- and USP19-ER-derived peptide sequences (coupled to the Keyhole Limpet Hemocyanin (KLH) antigen carrier) were used for immunization: H-CPEVAPTRTAPERFAP-NH_2_ and Ac-WVGPLPRGPTTPDEGC-NH_2_, respectively. Two rabbits were used, per peptide, and after 28 days, a total of three injections were performed. Preimmune, medium-bleed and large-bleed sera were collected. The enzyme-linked immunosorbent assay (ELISA) was performed by the company to analyze the levels of the antibodies, and the results are shown in Supplementary Fig. S10.

### Cloning, transfection, lentiviral infection and generation of stable cell lines

The primers and plasmids used for cloning are listed in Supplementary Table S1. Constructs containing the human USP19-ER and the enzymatically inactive mutant USP19-ER-C506S (CS) were a gift from Yihong Ye (Addgene plasmids 78597 and 78584) [26]. The plasmid containing human USP19-CY was constructed using the USP19-ER plasmid and a MYC-USP19 plasmid (without the TM domain), which was obtained from Novartis. The active site mutant USP19-CY-C506A (CA) was generated by site-directed mutagenesis. All these cDNAs were inserted into the pLV-CMV-IRES-PURO lentiviral vector. The human HA-TβRI-KDEL plasmid was constructed using pcDNA3-HA-TβRI (Addgene plasmid 80876) [36] and BFP-KDEL (a gift from Gia Voeltz, Addgene plasmid 49150) [37].

The lentivirus constructs were produced as previously described [23]. The USP19-CY and USP19-ER lentiviral short hairpin (sh)RNAs were obtained from Sigma (MISSION shRNA library), and the most effective shRNAs, namely, sh-USP19-CY (TRCN0000051713, 5′ GCGTGAT TTGATTCTGTTGTA-3′) and sh-USP19-ER (TRCN0000371018, 5′-GGCCATGCCTG CCTTTGTTGT-3′), were used. To generate stable cell lines, cells were infected with a 1:1 dilution of lentivirus in DMEM supplemented with 5 ng/mL of polybrene (Sigma), selected with puromycin for one week and subsequently cultured in the presence of puromycin to maintain selection pressure.

### ELISA

A coating solution with USP19-CY or USP19-ER peptide plus control carrier keyhole limpet hemocyanin (KLH) was added to a 96-well plate at a concentration of 100 ng/well and incubated for 16 h (h) at 4 °C. Then, the plate was blocked with 1 mg/mL bovine serum albumin (BSA) for 2 h at room temperature. Next, various dilutions (100x-218700x) of the preimmune serum and large-bleed were added into the designated wells and incubated for 2 h at room temperature. A horse radish peroxidase (HRP)-conjugated anti-rabbit IgG secondary antibody was diluted to 1:2500 in phosphate-buffered saline (PBS), added to the wells, and incubated for 2 h at room temperature. After adding 0.4 mg/mL o-phenylenediamine (OPD) and incubating for 20 min (min) at room temperature, 4 M H_2_SO_4_ was added to stop the reaction. The absorbance was measured at 492 nm within 30 min of adding the stop solution.

### Quantitative real-time-polymerase chain reaction (qRT-PCR)

Quantitative real-time-polymerase chain reaction (qRT-PCR) was performed as previously described [38]. The primer sequences that were used to measure the expression of specific genes are listed in Supplementary Table S1. All the target gene expression levels were normalized to that of *glyceraldehyde 3-phosphate dehydrogenase* (*GAPDH*). The results are shown as the mean ± SD with three biological replicates or as technical triplicates and are representative of three independent biological experiments.

### Ubiquitination, immunoprecipitation, immunoblotting and biotinylation

HEK293T cells were transfected with Myc-tagged constitutively active TβRI (Myc-caTβRI), HA-ubiquitin (HA-Ub) and the indicated constructs for 48 h and treated with 5 µM proteasome inhibitor MG132 (474787, Sigma) for 6 h. Next, the cells were lysed in 1% sodium dodecyl sulfate (SDS)-RIPA buffer (25 mM Tris–HCl, pH 7.4, 150 mM NaCl, 1% NP40, 0.5% sodium deoxycholate, and 1% SDS) supplemented with protease inhibitors (11836153001, Roche) and 10 mM NEM for 10 min on ice. The lysates were centrifuged at 11 × 10^3^ g for 10 min at 4 °C, and the protein concentrations were then measured using the DC protein assay (Pierce). Thereafter, the lysates were boiled for 5 min to eliminate the possibility of detecting the ubiquitination of coimmunoprecipitating proteins and diluted with 0.1% SDS in RIPA buffer. The lysates were then incubated with an anti-Myc antibody overnight, after which protein G-Sepharose (GE Healthcare Bio-Sciences AB) was added and incubated for 2 h at 4 °C (with rotation). After washing the beads with SDS-RIPA buffer, sample buffer was added to the beads, followed by immunoblotting analysis. For the immunoprecipitation assay, equal amounts of protein were incubated with anti-Flag agarose beads for 2 h at 4 °C (with rotation). Thereafter, the beads were washed five times with TNE buffer at 4 °C, and after adding sample buffer, they were boiled for 5 min. The immunoprecipitated proteins were then separated by SDS polyacrylamide gel electrophoresis (PAGE). Immunoblotting and the biotinylation analysis were performed as previously described [38, 39]. For the biotinylation, in brief, cells were biotinylated for 40 min at 4 °C, and then, the biotin-labeled cell surface proteins were precipitated with streptavidin beads and analyzed by immunoblotting. All the experiments were performed with biological triplicates, and representative results are shown.

### TAMRA (carboxytetramethylrhodamine)-ubiquitin-vinyl methyl ester (VME) probe assay

The TAMRA-VME probe assay was carried out as described previously [40]. Briefly, HEK293T cells transfected with USP19-CY-wt, USP19-CY-CA, USP19-ER-wt or USP19-ER-CS were lysed in TAMRA ABP buffer (50 mM Tris–HCl, pH 7.4, 250 mM sucrose, 5 mM MgCl_2_, 1 mM DTT, 0.5% zwitterionic surfactant CHAPS and 0.1% nonyl phenoxypolyethoxylethanol (NP40) supplemented with protease inhibitors. Then, the samples were sonicated for five cycles of 30 s (s) on and 30 s off on ice. Thereafter, the cell lysates were centrifuged at 16 × 10^3^ g for 15 min at 4 °C, and the supernatants were transferred to fresh Eppendorf tubes to measure the protein concentrations. The carboxytetramethylrhodamine ubiquitin-vinyl methyl ester (TAMRA-Ub-VME) probe (UbiQ-050; UbiQ) was used at a concentration of 1 µM to label 25 µg of protein extracts in a total volume of 25 µL for 30 min at room temperature. The labeling reactions were terminated by the addition of sample buffer and heating to 100 °C for 10 min. The labeled proteins were separated by NuPAGE 4–12% Bis–Tris protein gels (WG1402BOX; Invitrogen), and the fluorescence signals were detected using the Typhoon FLA 9500 Molecular Imager (GE Healthcare) at an excitation wavelength of 550 nm and an emission wavelength of 590 nm.

### Transcriptional response assay

The SMAD3/4-dependent CAGA_12_-transcriptional luciferase reporter assay was performed as described previously [38]. Briefly, HEK293T cells were transfected with the CAGA_12_-luc reporter, β-galactosidase encoding plasmids and the indicated plasmid using PEI for 24 h. Then, the cells were serum starved for 8 h and treated with or without TGF-β (1 ng/ml) overnight. CAGA_12_-mediated transcriptional activity was normalized to β-galactosidase expression. All the experiments were performed in biological triplicates, and representative results are shown.

### Dynamic measurement of RFP-vimentin expression

A549-VIM-RFP cells (in which the red fluorescent protein coding region is cloned in frame in the endogenous Vimentin gene locus [41]) were used to analyze the EMT process by measuring the dynamic changes in red fluorescent protein (RFP)-tagged vimentin expression. Cells that were transfected with the indicated plasmids (pLKO-EV, sh-CY or sh-ER) were cultured in a 96-well plate in the IncuCyte live cell imaging system and treated with vehicle control or TGF-β (2.5 ng/ml) for the indicated time points. The RFP signals were captured every 4 h over a period of 58 h using a 10 × objective. Then, the RFP-vimentin intensity was analyzed by IncuCyte software and normalized to the RFP signals observed at 0 h in each group. All the experiments were performed in biological triplicates, and representative results are shown.

### IncuCyte and transwell migration assay

MDA-MB-231 cells that were transfected with the indicated plasmids (pLKO-EV, sh-CY or sh-ER) were seeded in a IncuCyte 96-well Essen ImageLock plate (4379, Essen BioScience) and scratched using the IncuCyte WoundMaker (Essen BioScience). The scratched cells were washed with PBS and then cultured in the IncuCyte live cell imaging system. Images were acquired every 2 h over a 14–20 h period using a 10 × objective. The relative wound size in each well was analyzed by IncuCyte cell migration software.

Transwell assays were performed in 24-well invasion chambers with an 8.0 µm polyethylene terephthalate membrane (354483, Corning). MDA-MB-231 cells overexpressing USP19-ER-wt or USP19-ER-CS were serum starved overnight and then, seeded into the Transwell inserts, and DMEM supplemented with 10% FBS was added to the lower part of the chamber. The cells inside the chamber were carefully removed by a cotton tip that had been moistened with PBS, and the migrated cells were fixed in 4% paraformaldehyde (PFA, 28908, Thermo Fisher Scientific) for 10 min. These migrated cells were stained with 0.5% crystal violet for 30 min. Five random fields were selected and photographed for each condition, and the number of cells was counted using ImageJ. All the experiments were performed in biological triplicates, and representative results are shown.

### Nano-Glo HiBiT lytic detection assay

MDA-MB-231 cells stably expressing green fluorescent protein (GFP) were generated as previously described [42]. The HiBiT tag, which is a small 11-amino acid peptide [43], was knocked in using CRISPR/Cas9 technology at the endogenous TβRI locus, resulting in a TβRI in which the HiBiT sequence is inserted at the carboxy terminus of the signal peptide. This cell line allows for the specific detection and quantification of TβRI expression at the cell surface by the addition of large BiT (LgBiT) to the cell medium. The HiBiT-TβRI cell line was infected with pLV-EV, USP19-ER-wt or USP19-ER-CS lentivirus and then, seeded into a 384-well plate (781098, Greiner Bio-one). After the cells were allowed to adhere overnight, the medium was removed and replaced with the PBS/LgBiT/NanoGlo substrate mixture from the NanoGlo-HiBiT Detection kit (N2420, Promega). The cells were incubated with the substrate mixture for 15 min, and the signals were measured using a VICTOR multilabel plate reader (2030-0050, PerkinElmer). Thereafter, the plate was imaged in the IncuCyte live cell imaging system to measure the GFP intensity, which is a proxy for the number of live cells. The NanoGlo signals were normalized to the GFP intensity.

### Cell viability assay

The A549-VIM-RFP cells were seeded in 96-well plates and treated with various concentration of herboxidiene for 24 h. Then, cell viability was determined using CellTiter 96 Aqueous One Solution Cell Proliferation Assay (G3582, Promega) containing tetrazolium compound 3-[4,5-dimethylthiazol-2-yl]-5-(3-carboxymethoxyphenyl)-2-(4-sulfophenyl)-2*H*-tetrazolium (MTS) to measure the mitochondrial activity according to the manufacturer’s protocol. Twenty microliters of MTS were added to each well, incubated at 37 °C for 1 h, and then, the absorbance was measured at 490 nm with a luminometer (2030-0050, PerkinElmer).

### Zebrafish extravasation assay

Zebrafish extravasation assays were performed as previously described [44]. The experiments were carried out according to the standard guidelines that were approved by the local Institutional Committee for Animal Welfare of Leiden University. The fish were fixed with 4% paraformaldehyde (PFA) four days after injection with mCherry-labeled MDA-MB-231 cells into the Duct of Cuvier and imaged by inverted SP5 confocal microscopy (Leica Microsystems). The numbers of cancer cells that had invaded into the avascular tail fin area, which is rich in collagen, were counted (Supplementary Fig. S5E). The experiments were repeated twice in biologically independent experiments, and at least 25 injected embryos were included for quantification.

### Formalin-fixed cell line plug preparation and immunofluorescence staining

To prepare the formalin-fixed cell line plugs for incorporation into paraffin blocks, we used ultralow gelling temperature (ULGT) agarose (Agarose type IX-A, Sigma) as the resuspension medium and a standard agarose (Agarose type I-A, Sigma) as the re-embedding medium. First, HEK293T cells with or without USP19-CY-wt overexpression were fixed with 10% formalin for 3 h at 4 °C and then, centrifuged for 30 s at 12 × 10^3^ g. The supernatants were discarded. Then, the cells were resuspended in 50 µL of 3% ULGT agarose solution and centrifuged for 30 s at 12 × 10^3^ g. After removal of the supernatants, the compact agarose cell pellets were solidified for 10 min at 4 °C. Thereafter, the cell pellets were transferred to the cap of an Eppendorf tube, which was further filled with the standard agarose solution. After solidification of the standard agarose gel at room temperature for 2 min, the agarose cell pellets were placed in tissue cassettes, subjected to routine tissue processing using an automated tissue processor machine and embedded in paraffin. Then, the cell line plugs were sectioned and mounted on a slide for immunofluorescence staining, which was performed using the same protocol as the IF staining with patient tissues.

The formalin-fixed paraffin-embedded microarrays of breast cancer tissues were purchased and included matched breast cancer and cancer adjacent tissues (BR804b, Biomax), and breast cancer tissues of different stages (IIA, IIB, IIIA and IIIB stages, BC081116e, Biomax). Both tissue arrays were used for immunofluorescence staining. The tissue microarrays were incubated overnight at 37 °C and then, for 2 h at 60 °C until the paraffin melted. The slides were then incubated in a histoclear bath for 7 min three times. Thereafter, the slides were rehydrated in fresh absolute ethanol for 7 min twice and transferred once through 90%, 70%, and 50% ethanol solutions, for 3 min each and washed twice with Milli-Q water for 7 min each. The slides were boiled in an antigen unmasking buffer (1.5 M Tris, pH 8.0, 0.5 M EDTA, 10% Tween-20) for 35 min using a pressure cooker. The tissue microarrays were then washed twice with Milli-Q water for 5 min and once with PBS. Thereafter, the slides were blocked using 1% BSA diluted in PBS/0.1% Tween for 30 min and incubated with the primary USP19-CY antibody at a 1:100 dilution in PBS/Tween containing 1% BSA overnight at 4 °C. Thereafter, the Alexa Fluor 555-conjugated secondary antibody, which was diluted to 1:250 in PBS/Tween/BSA, was added to the tissue arrays and incubated for 2 h at room temperature. Subsequently, the slides were washed twice with PBS/Tween. The slides were then incubated with DAPI (diluted 1:1000 in PBS) for 10 min and washed twice with PBS/Tween. Prolong Gold antifade Mountant (P36930, Thermo) was used to mount the slides. The stained tissue arrays were imaged using a ZEISS Axio Scan Z1 slide scanner. The percent USP19-CY expression in each of the tissues in the arrays was analyzed using QuPath software. The analysis of the tissue sections was performed in an unbiased blinded manner.

IF staining of cell lines was performed as described previously [38]. The experiments were performed with biological triplicates, and representative results are shown.

### Statistical analysis

Statistical analyses were performed using Student’s unpaired *t* test using Prism 8 software (GraphPad La Jolla, CA) or as indicated in the legends. All the data are expressed as the mean ± SD with three biological replicates or as indicated in the legends. The p value is indicated by asterisks in the figures: *P ≤ 0.05; **P < 0.01; ***P < 0.001; ****P < 0.0001. *P* ≤ 0.05 was considered statistically significant.

## Results

### USP19-CY promotes TGF-β signaling, while USP19-ER exerts the opposite effect

**Fig. 1:**
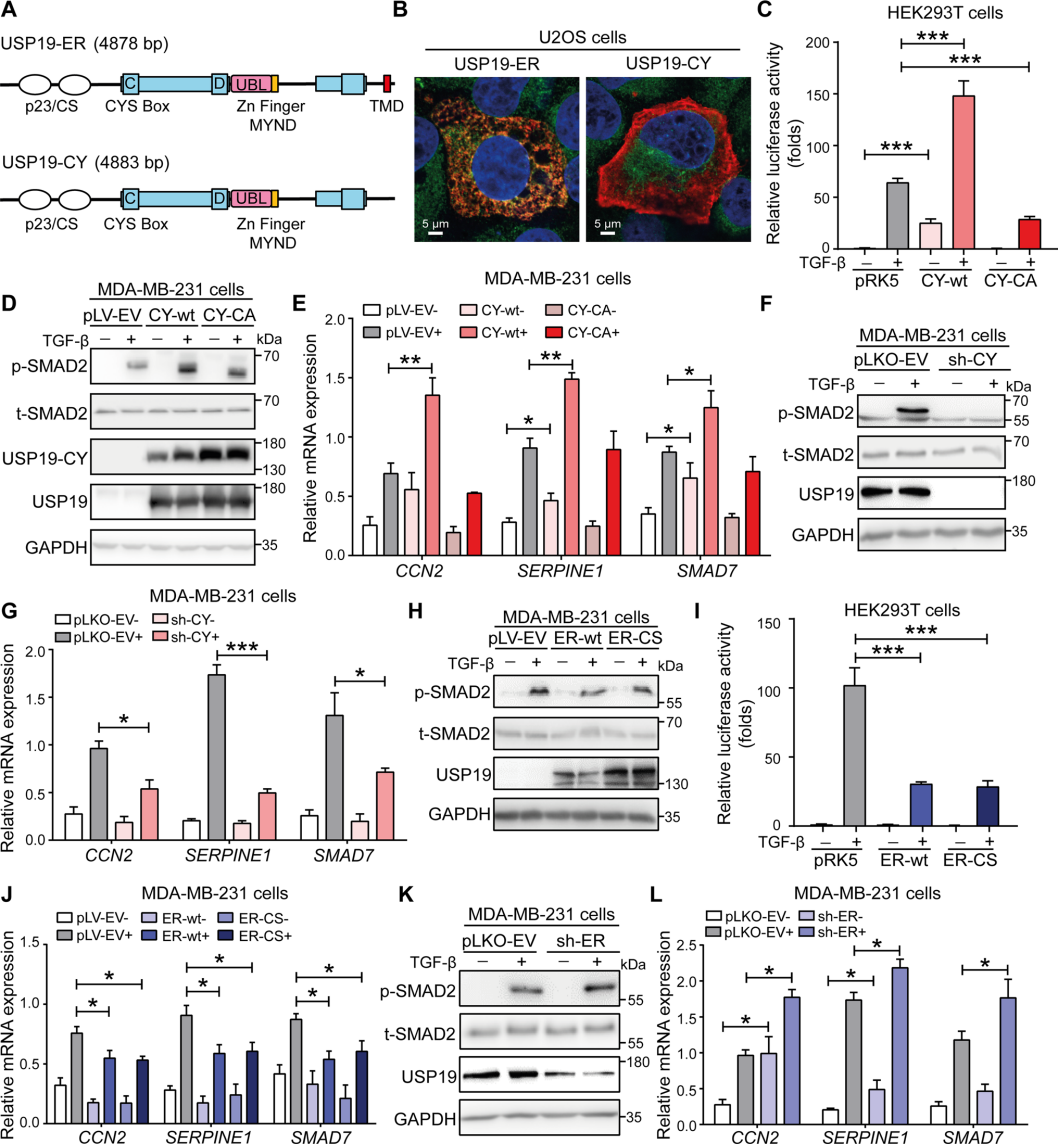
The USP19 cytosolic isoform (USP19-CY) promotes TGF-β signaling; conversely, the endoplasmic reticulum (ER)-localized USP19 isoform (USP19-ER) inhibits this TGF-β pathway. **A** Schematic diagram showing the depicting USP19-ER and USP19-CY isoforms with common structural domains, including a catalytic domain bearing the essential cysteine (C), aspartic acid (D) and histidine triad of amino acid residues required for catalysis, and unique C-terminal regions. The C-terminal transmembrane domain (TMD) causes the ER localization of the USP19-ER isoform. The catalytic domain also bears a putative ubiquitin-like (UBL) domain as well as a MYND Zn finger domain that is involved in protein‒protein interactions. **B** Immunofluorescence analysis of the localization of USP19 (red) and calnexin (green) in U2OS cells transfected with FLAG-tagged wild-type USP19-CY and USP19-ER expression plasmids. Nuclei were counterstained with 4,6-diamidino-2-phenylindole (DAPI, blue). Images were captured with confocal microscopy. Scale bar = 5 μm. **C** Effect of USP19-CY-wt or USP19-CY-CA on the SMAD3-dependent CAGA_12_-luciferase transcriptional response induced by TGF-β (2.5 ng/mL; overnight treatment) in HEK293T cells. The data are expressed as the mean ± SD, *n* = 3 (biological replicates). ***P < 0.001, based on unpaired Student’s *t* test. **D** Immunoblotting analysis of the p-SMAD2, total (t)-SMAD2, USP19-CY and total USP19 levels in MDA-MB-231 cells that were infected with empty vector (pLV-EV), wild-type USP19-CY (CY-wt) or USP19-CY enzyme inactive mutant (CY-CA) lentivirus after stimulation with vehicle control or TGF-β (2.5 ng/mL) for 1 h. GAPDH, loading control. **E** qRT‒PCR analysis of TGF-β target genes, i.e., *CCN2*, *SERPINE1* and *SMAD7*, in MDA-MB-231 cells stably infected with pLV-EV, CY-wt, or CY-CA in the presence of vehicle control or TGF-β (2.5 ng/mL) for 6 h. The data are expressed as the mean ± SD, *n* = 3 (biological replicates). *P ≤ 0.05, **P < 0.01, based on unpaired Student’s *t* test. **F** Western blotting analysis of the p-SMAD2, t-SMAD2 and USP19 levels in MDA-MB-231 cells with or without shRNA-mediated specific knockdown of USP19-CY (sh-CY) treated with vehicle control or TGF-β (2.5 ng/mL) for 1 h. GAPDH, loading control. **G** Expression levels of the TGF-β target genes, *CCN2*, *SERPINE1* and *SMAD7* in pLKO-EV control or USP19-CY-deficient MDA-MB-231 cells treated with vehicle control or TGF-β (2.5 ng/mL) for 6 h. The data are expressed as the mean ± SD, *n* = 3 (biological replicates). *P ≤ 0.05, ***P < 0.001, based on unpaired Student’s *t* test. **H** Immunoblotting analysis of the p-SMAD2, t-SMAD2 and USP19 levels in MDA-MB-231 cells infected with pLV-EV, wild-type USP19-ER (ER-wt) and USP19-ER enzyme inactive mutant (ER-CS) and treated with vehicle control or TGF-β (2.5 ng/mL) for 1 h. GAPDH, loading control. **I** Measurement of the SMAD3-dependent CAGA_12_-luciferase transcriptional activity induced by overnight treatment with TGF-β (2.5 ng/mL) in HEK293T cells that were transfected with ER-wt or ER-CS or pLV-EV expression plasmids. The data are expressed as the mean ± SD, *n* = 3 (biological replicates). ***P < 0.001, based on unpaired Student’s *t* test. **J** qRT‒PCR analysis of the expression of TGF-β target genes, i.e., *CCN2*, *SERPINE1* and *SMAD7*, in MDA-MB-231 cells stably infected with pLV-EV, ER-wt or ER-CS in the presence of vehicle control or TGF-β (2.5 ng/mL) for 6 h. The data are expressed as the mean ± SD, *n* = 3 (biological replicates). *P ≤ 0.05, based on unpaired Student’s *t* test. **K** Immunoblotting of the p-SMAD2, t-SMAD2 and USP19 levels in MDA-MB-231 cells with or without shRNA-mediated knockdown of USP19-ER (sh-ER) treated with vehicle control or TGF-β (2.5 ng/mL) for 1 h. GAPDH, loading control. **L** Expression levels of TGF-β target genes, i.e., *CCN2*, *SERPINE1* and *SMAD7*, in PLKV-EV control or USP19-ER-deficient MDA-MB-231 cells treated with vehicle control or TGF-β (2.5 ng/mL) for 6 h. The data are expressed as the mean ± SD, *n* = 3 (biological replicates). *P ≤ 0.05, ***P < 0.001, based on unpaired Student’s *t* test

USP19 is expressed in cells as two major distinct isoforms that are produced by the alternative splicing of the 3’ terminal exon (Fig. S1) [27]. The USP19-ER isoform contains the TMD that targets USP19 to the ER membrane, where its active site faces the cytosol. This TMD is not present in the USP19-CY isoform, which localizes to the cytoplasm (Fig. 1A). Using immunofluorescence staining of U2OS cells, we confirmed previous observations that USP19-ER is an ER-anchored protein that colocalizes with the ER protein calnexin; conversely, USP19-CY showed cytoplasmatic and plasma membrane localization and did not colocalize with calnexin (Fig. 1B, Supplementary Fig. S2C). For the specific detection and depletion of the USP19 splice variants, we designed primers, shRNAs and antibodies based on the different cDNA sequences and encoded C-terminal sequences of USP19-ER and USP19-CY (Supplementary Fig. S2A, S2B). USP19 is a member of the ubiquitin-specific protease family, and we confirmed that the USP19-ER and USP19-CY variants have deubiquitinating activity using the TAMRA-VME probe assay. Both ER-wt and CY-wt, but not the inactive ER-CS and CY-CA mutants, were capable of covalently interacting with the TAMRA-VME probe (Supplementary Fig. S2D). Then, we investigated the role of USP19-CY in regulating TGF-β signaling. Consistent with our previous report [23], we found that ectopic expression of USP19-CY-wt promoted a TGF-β-induced SMAD3/4-dependent transcriptional response (Fig. 1C). Interestingly, overexpression of the USP19-CY-CA mutant significantly inhibited this TGF-β-induced response (Fig. 1C). To further validate this result, we generated MDA-MB-231 cells that stably express FLAG-tagged USP19-CY-wt or USP19-CY-CA. The ectopic expression of USP19-CY was confirmed at the mRNA and protein levels (Supplementary Fig. S3A and Fig. 1D). MDA-MB-231 cells expressing USP19-CY-wt exhibited significantly enhanced TGF-β-induced SMAD2 phosphorylation; conversely, ectopic expression of the USP19-CY-CA mutant failed to upregulate the TGF-β-induced p-SMAD2 levels (Fig. 1D**, **Supplementary Fig. S3B). Moreover, the USP19-CY-wt-induced upregulation of p-SMAD2 was also observed in HEK293T cells transfected with the control plasmid (pRK5), CY-wt or CY-CA after stimulation with TGF-β (Supplementary Fig. S3C). Consistent with this notion, the ectopic expression of CY-wt in MDA-MB-231 cells significantly enhanced the transcription levels of TGF-β target genes, including *CCN2* (encodes the CTGF protein), *SERPINE1* (encodes the PAI1 protein) and *SMAD7* (encodes the SMAD7 protein), after TGF-β treatment for 6 h, but the ectopic expression of the CY-CA mutant did not exert this effect (Fig. 1E). Thus, USP19-CY promotes TGF-β/SMAD signaling in a DUB-dependent manner. Moreover, MDA-MB-231 cells in which USP19-CY mRNA and protein were specifically depleted (Fig. 1F, Supplementary Fig. S3D), showed strong decreases in the TGF-β-induced p-SMAD2 levels (Fig. 1F, Supplementary Fig. S3E). This inhibition of SMAD2 phosphorylation was also observed in other cell lines lacking USP19-CY, including MCF10A-Ras cells and A549-VIM-RFP cells (Supplementary Fig. S3F–I). In addition, after shRNA-mediated USP19-CY depletion in MDA-MB-231 cells, MCF10A-Ras cells and A549-VIM-RFP cells, the TGF-β-mediated induction of the expression of target genes, including *CCN2*, *SERPINE1* and *SMAD7,* were decreased (Fig. 1G, Supplementary Fig. S3J, S3K).

Next, similar assays were performed to investigate the role of USP19-ER in TGF-β signaling. After validating the ectopic expression efficiency of ER-wt and ER-CS mutants in MDA-MB-231 cells by qRT‒PCR analysis (Supplementary Fig. S4A), we found a significant decrease in the p-SMAD2 levels in cells with ER-wt or ER-CS after treatment with TGF-β (Fig. 1H, Supplementary Fig. S4B). This inhibition of TGF-β signaling was also observed in HEK293T cells transfected with ER-wt or ER-CS (Supplementary Fig. S4C). Furthermore, the overexpression of ER-wt or ER-CS strongly suppressed the TGF-β-induced SMAD-dependent luciferase reporter transcription activity and the transcription levels of TGF-β target genes, i.e., *CCN2*, *SERPINE1* and *SMAD7* (Fig. 1I, J). Thus, these results indicate that USP19-ER, in contrast to USP19-CY, inhibits TGF-β signaling and that the catalytic activity of USP19-ER is not needed in this process. Consistent with this notion, the specific depletion of USP19-ER in MDA-MB-231 or A549-VIM-RFP cells increased the TGF-β-induced p-SMAD2 levels (Fig. 1K, Supplementary Fig. S4D–G). Consistent with this finding, knockdown of USP19-ER upregulated the expression levels of TGF-β target genes, including *CCN2*, *SERPINE1* and *SMAD7*, in MDA-MB-231 cells and A549-VIM-RFP cells (Fig. 1L, Supplementary Fig. S4H). Taken together, these results suggest that the catalytic activity of USP19-CY is required to promote TGF-β signaling, while USP19-ER inhibits TGF-β signaling in a DUB activity-independent manner.

### The USP19-ER isoform inhibits TGF-β-induced EMT and cell migration

**Fig. 2:**
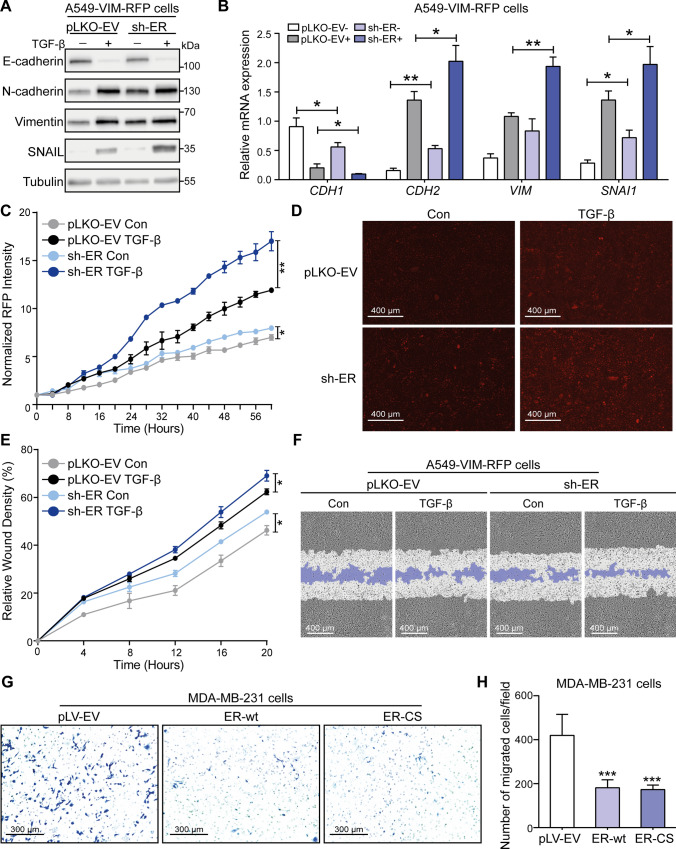
USP19-ER isoform inhibits TGF-β-induced EMT, and cell migration. **A** Immunoblotting analysis of the expression levels of the epithelial marker E-cadherin, mesenchymal markers N-cadherin, vimentin and SNAIL, and USP19 in USP19-ER-deficient A549-VIM-RFP cells that were treated with vehicle control or TGF-β (2.5 ng/mL) for 2 days. Tubulin, was used as a loading control. **B** qRT‒PCR analysis of the expression of the EMT marker genes *CDH1* (encodes the E-cadherin protein), *CDH2* (encodes the N-cadherin protein), *VIM* (encodes the vimentin protein) and *SNAI1* (encodes the SNAIL protein) in A549-VIM-RFP cells with pLKO-EV or USP19-ER shRNA in the presence of TGF-β (2.5 ng/mL) for 2 days. The data are expressed as the mean ± SD, *n* = 3 (biological replicates). *P ≤ 0.05, **P < 0.01, based on unpaired Student’s *t* test. **C** Effect of USP19-ER knockdown on vimentin expression in A549-VIM-RFP cells in response to TGF-β (2.5 ng/mL) treatment for the indicated times. The expression of RFP-conjugated vimentin expression over time was measured by IncuCyte. Red staining intensity was normalized to the intensity at 0 h and expressed as the mean ± SD, *n* = 3 (biological replicates). **P < 0.01, based on two-way ANOVA. **D** Representative images of RFP-vimentin expression at the end time point (58 h) in A549-VIM-RFP cells with pLKO-EV or sh-ER. Scale bar = 400 μm. **E** Real-time scratch assay results, as analyzed by IncuCyte, in pLKO-EV control or USP19-ER-depleted A549-VIM-RFP cells treated with vehicle control or TGF-β (2.5 ng/mL) for the indicated times. The relative wound density (closure) is presented as the mean ± SD, *n* = 3 (biological replicates). *P ≤ 0.05, based on two-way ANOVA. **F** Representative images of a scratch wound in pLKO-EV control or USP19-ER-deficient A549-VIM-RFP cells that were treated with vehicle control or TGF-β (2.5 ng/mL) at the end time point. The region of the original scratch is indicated in white, and the remaining scratch area is indicated in purple. Scale bar = 400 μm. **G** Crystal violet staining of MDA-MB-231 cells stably infected with the pLV-EV, ER-wt or ER-CS lentivirus after the Transwell migration assay. Scale bar = 300 μm. **H** Quantification of the migrated MDA-MB-231 cells stably expressing pLV-EV, ER-wt and ER-CS in the Transwell assay. The number of migrated cells per field is shown as the mean ± SD, *n* = 5 (biological replicates). ***P < 0.001, based on unpaired Student’s *t* test

To examine the effect of abnormal USP19-ER expression on EMT, we analyzed the changes in EMT markers expression in A549-VIM-RFP cells lacking USP19-ER. ShRNA-mediated knockdown of USP19-ER slightly decreased the expression of the epithelial marker E-cadherin but significantly increased the expression of mesenchymal markers, including N-cadherin, vimentin and SNAIL, in the presence or absence of exogenous TGF-β (Fig. 2A, Supplementary Fig. S5A). Consistent with the immunoblotting analysis, the depletion of USP19-ER downregulated the transcription level of the epithelial marker *CDH1* (encodes the E-cadherin protein), while promoting the mRNA expression levels of the mesenchymal markers *CDH2* (encodes the N-cadherin protein), *VIM* (encodes the vimentin protein) and *SNAI1* (encodes the SNAIL protein) (Fig. 2B). The USP19-ER knockdown-mediated promotion of TGF-β-induced EMT was further confirmed by the dynamic increase in RFP-tagged vimentin expression (Fig. 2C, D). We next investigated the role of USP19-ER in cell migration. The shRNA-mediated depletion of USP19-ER significantly enhanced both basal and TGF-β-induced A549 cell migration in the scratch assay (Fig. 2E, F). Similarly, using a Transwell assay, we found that fewer MDA-MB-231 cells that stably overexpressed ER-wt or ER-CS had migrated than control cells (Fig. 2G, H). Collectively, these results indicate the negative regulatory role of the USP19-ER isoform (independent of its DUB activity) in TGF-β-induced EMT and cell migration.

### The USP19-CY isoform enhances TGF-β-induced EMT, cell migration in vitro and invasion in vivo

**Fig. 3:**
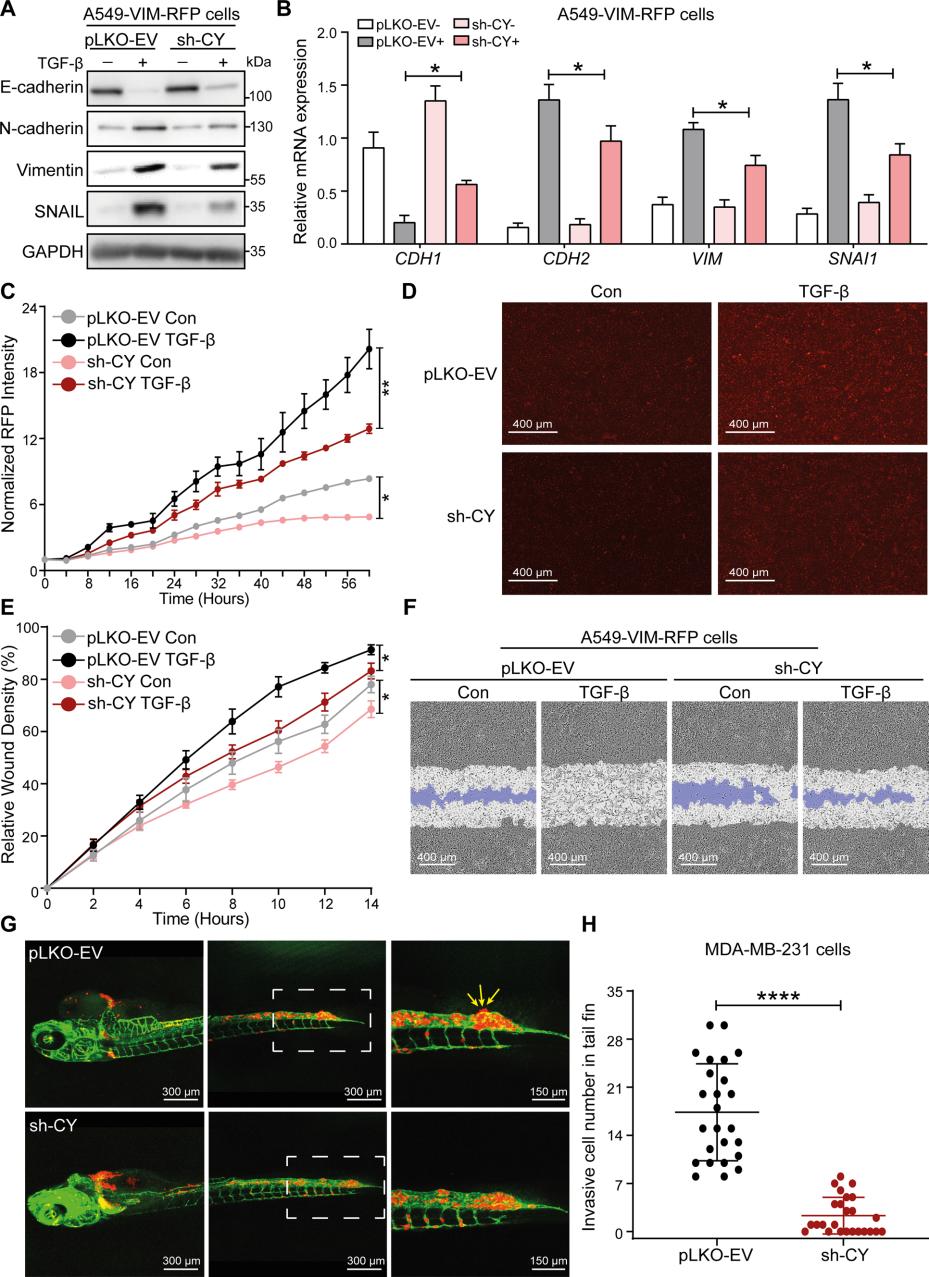
USP19-CY promotes TGF-β-induced EMT, cell migration and invasion. **A** Western blotting analysis of epithelial marker E-cadherin, mesenchymal markers N-cadherin, vimentin and SNAIL, and USP19 expression levels in A549-VIM-RFP cells without (pLKO-EV) or with USP19-CY knockdown that were treated with vehicle control or TGF-β (2.5 ng/mL) for 2 days. Tubulin was used as the loading control. **B** qRT‒PCR analysis of the expression of the EMT marker genes *CDH1*, *CDH2*, *VIM* and *SNAI1* in A549-VIM-RFP cells infected with pLKO-EV or sh-CY in the absence or presence of TGF-β (2.5 ng/mL) for 2 days. The data are expressed as the mean ± SD, *n* = 3 (biological replicates). *P ≤ 0.05, based on unpaired Student’s *t* test. **C** Effect of USP19-CY depletion on vimentin expression in A549-VIM-RFP cells treated with or without TGF-β (2.5 ng/mL) for the indicated times. Time course of RFP-conjugated vimentin expression levels as measured by IncuCyte. Red staining intensity was normalized to the intensity at 0 h and expressed as the mean ± SD, *n* = 3 (biological replicates). *P ≤ 0.05, **P < 0.01, based on two-way ANOVA. **D** Representative images of RFP-vimentin expression in A549-VIM-RFP cells with pLKO-EV or sh-CY at the end time point (58 h). Scale bar = 400 μm. **(E)** Real-time scratch assay results, as analyzed by IncuCyte, in A549-VIM-RFP control cells (pLKO-EV) or with USP19-CY-knockdown cells treated with vehicle control or TGF-β (2.5 ng/mL) for the indicated times. The relative wound density (closure) is presented as the mean ± SD, *n* = 3 (biological replicates). *P ≤ 0.05, based on based on two-way ANOVA. **F** Representative images of scratch wounds in pLKO-EV control or USP19-CY-depleted A549-VIM-RFP cells that were treated with vehicle control or TGF-β (2.5 ng/mL) at the end time point. The area of the original scratch is indicated in white, and the open area of the scratch is indicated in purple. Scale bar = 400 μm. **G** mCherry-labeled MDA-MB-231 cells with pLKO-EV and sh-CY were injected into ducts of Cuvier of zebrafish embryos. Representative images with magnified pictures (outlined with a dashed square) of invasive cells were captured 4 days after injection by confocal microscopy. Scale bar = 300 μm or 150 μm. Extravasated cells in the avascular collagen-rich tail fin area are indicated with three arrows. **H** Quantification of the number of invasive cells in the tail fins of 25 embryos for each group. The data are expressed as the mean ± SD, *n* = 2 (biological replicates). ****P < 0.0001, based on unpaired Student’s *t* test

To gain insight into the role of USP19-CY in TGF-β-induced EMT, we first examined the effect of its specific depletion on TGF-β-induced EMT marker expression. The shRNA-mediated knockdown of USP19-CY significantly increased the expression of the epithelial marker E-cadherin but inhibited the expression of mesenchymal markers, i.e., N-cadherin, vimentin and SNAIL, both at the mRNA and protein levels in A549 cells treated with TGF-β (Fig. 3A, B, Supplementary Fig. S5B). Similarly, shRNA-mediated knockdown of USP19-CY (sh-CY) upregulated E-cadherin expression and decreased N-cadherin and vimentin expression in MCF10A-Ras cells treated with TGF-β (Supplementary Fig. S5C). Moreover, we analyzed the dynamic changes in the expression of RFP-labeled vimentin using IncuCyte and found that USP19-CY depletion inhibited vimentin expression both in control cells and in TGF-β-treated A549-VIM-RFP cells (Fig. 3C, D). In addition, the effect of USP19-CY knockdown on cell migration was examined by a wound healing assay; USP19-CY depletion significantly downregulated the basal and TGF-β-induced migratory abilities of A549 cells (Fig. 3E, F). To further investigate whether USP19-CY affects cell invasion, we injected mCherry-labeled MDA-MB-231 cells with pLKO-EV and sh-CY (knockdown efficiency was validated by western blotting analysis as shown in Supplementary Fig. S5D) into the ducts of Cuvier (Doc) of zebrafish embryos (Supplementary Fig. S5E). A significantly lower number of extravascular MDA-MB-231 cells in the tail fin was observed in the USP19-CY-depleted group than in the control group four days after injection (Fig. 3G, H). These results suggest that USP19-CY promotes TGF-β-induced EMT, as well as basal and TGF-β-mediated cell migration and invasion.

### The USP19-ER isoform interacts with and sequesters TβRI in the ER, resulting in decreased expression of TβRI at the cell membrane

**Fig. 4:**
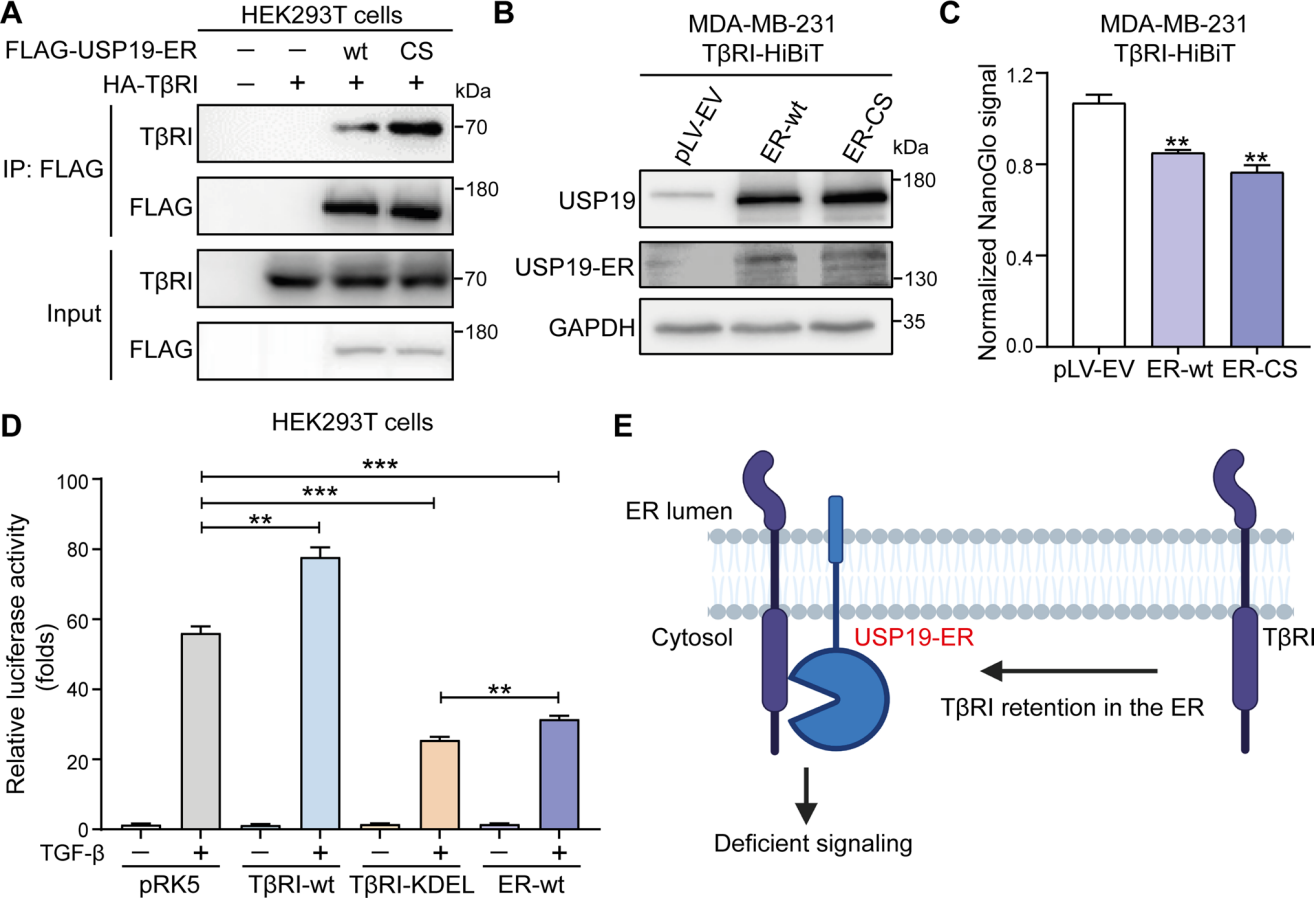
USP19-ER binds to TβRI and sequesters TβRI in the ER to decrease its expression on the plasma membrane. **A** The interaction of USP19-ER and TβRI was analyzed by IP of FLAG-tagged USP19-ER (wt or CS mutant) and immunoblotting for TβRI in HEK293T cells. **B** Western blotting analysis of the expression levels of USP19 and USP19-ER in MDA-MB-231 cells in which TβRI was endogenously tagged with HiBiT. **C** Measurement of TβRI-HiBiT expression by the detection of NanoGlo signals in MDA-MB-231 cells with pLV-EV, USP19-ER-wt and USP19-ER-CS. The results were normalized to the GFP intensity of the cells and expressed as the mean ± SD of three biological replicates. **P < 0.01, based on unpaired Student’s *t* test. **D** Effect of TβRI-wt, TβRI containing the KDEL sequence at the carboxy (C)-terminus (TβRI-KDEL) or USP19-ER-wt on the CAGA_12_-luciferase transcriptional response induced by overnight treatment with TGF-β (2.5 ng/mL) in HEK293T cells. The data are expressed as the mean ± SD, *n* = 3 (biological replicates). **P < 0.01, ***P < 0.001, based on unpaired Student’s *t* test. **E** Summary diagram showing USP19-ER-mediated inhibition of TGF-β signaling by sequestering TβRI in the ER and decreasing the amount of TβRI on the cell membrane

Next, we investigated the mechanism by which USP19-ER inhibits TGF-β/SMAD signaling. As USP19-ER inhibits TGF-β-induced SMAD2 phosphorylation, we hypothesized that USP19-ER may interact with the upstream activator of p-SMAD2, i.e., TβRI. We therefore performed an immunoprecipitation of USP19-ER followed by Western blotting for TβRI in HEK293T cells that were transfected with FLAG-tagged USP19-ER-wt or USP19-ER-CS and HA-tagged TβRI. We observed that TβRI interacted with both USP19-ER-wt and USP19-ER-CS (Fig. 4A). Consistently, the interaction was also observed cells between endogenous TβRI and ectopically expressed USP19-ER-wt or USP19-ER-CS in MDA-MB-231 cells (Supplementary Fig. S6A). To further validate these results, we analyzed the cell surface expression of endogenous TβRI that was epitope tagged with a HiBiT sequence in MDA-MB-231 cells. We infected these cells with pLV-EV, USP19-ER-wt or USP19-ER-CS lentivirus. The overexpression of USP19-ER (wt and CS) was confirmed by Western blotting using USP19 and USP19-ER antibodies (Fig. 4B). The quantification of normalized NanoGlo signals showed lower cell surface TβRI expression in MDA-MB-231 cells expressing ER-wt and ER-CS compared to the empty vector group (Fig. 4C). In addition, we observed a significant increase in cell surface TβRI levels in MDA-MB-231 cells lacking USP19-ER compared to control cells using biotin-labeling of cell surface proteins followed by immunoprecipitation of TβRI (Supplementary Fig. S6B). Since USP19-ER is a tail-anchored DUB that localizes to the ER [27], we hypothesized that USP19-ER may sequester TβRI in the ER by interacting with it and thereby interfere with its transportation to the cell membrane. To validate this hypothesis, we generated TβRI construct with a C-terminal KDEL sequence to target it to the ER [45] and performed a CAGA_12_-luciferase reporter assay in HEK293T cells that were transfected with empty control (pRK5), wild-type TβRI (TβRI-wt), TβRI-KDEL or USP19-ER-wt. Consistent with the expectation, cells transfected with the TβRI-KDEL plasmid showed a significant decrease in TGF-β/SMAD-induced luciferase activity compared to the control cells and cells transfected with the TβRI-wt plasmid, and the luciferase activity in the TβRI-KDEL group was comparable to that in the USP19-ER-wt group (Fig. 4D). Taken together, these findings indicate that USP19-ER inhibits TGF-β signaling in a catalytic activity-independent manner by restraining TβRI in the ER (Fig. 4E).

### The USP19-CY isoform binds to TβRI, protects it from ubiquitination and increases its stability

**Fig. 5:**
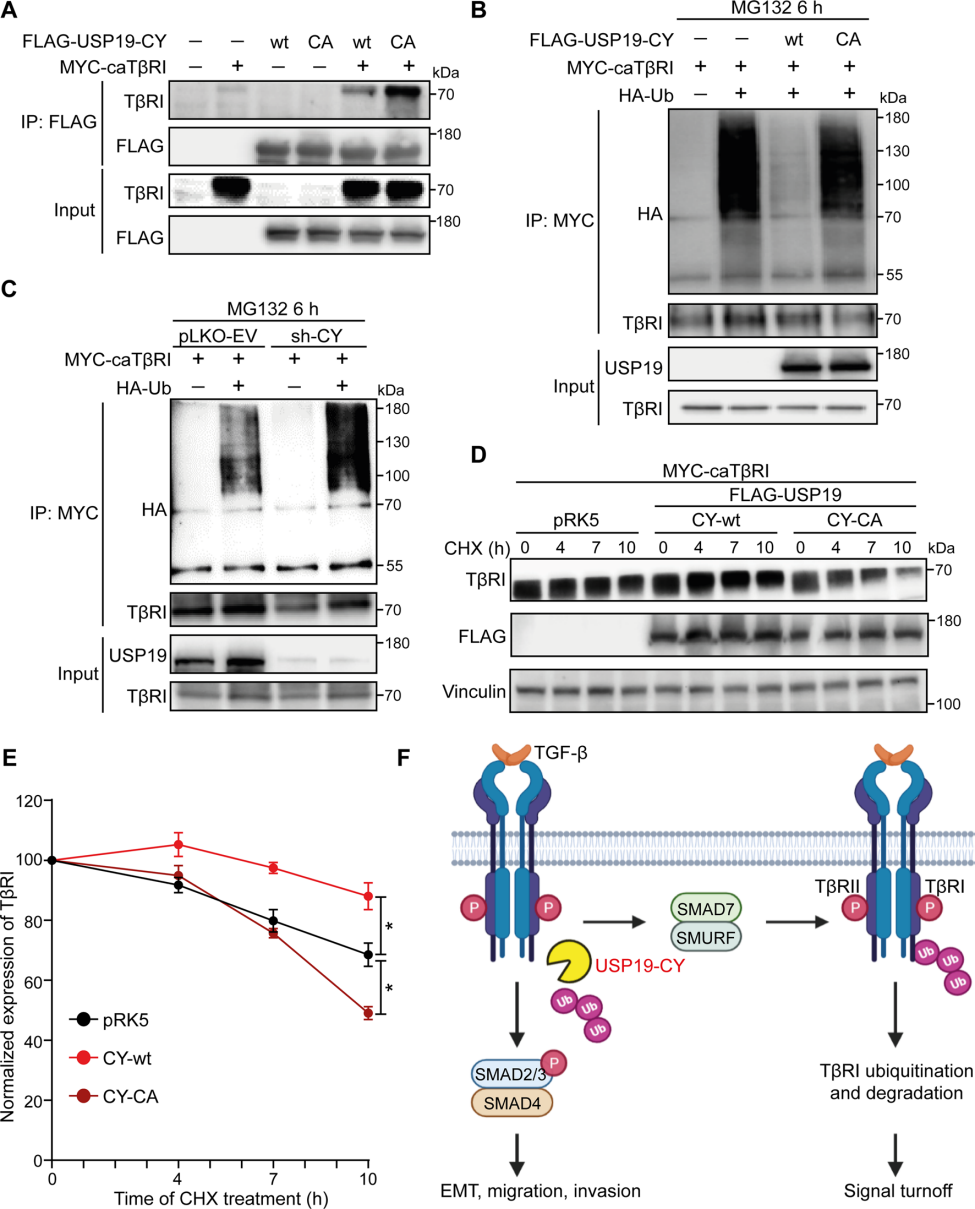
USP19-CY binds to TβRI and inhibits the ubiquitination and degradation of TβRI. **A** The interaction of USP19-CY and TβRI was analyzed by immunoprecipitation (IP) of FLAG-tagged USP19-CY (wt or CA mutant) and immunoblotting for TβRI in HEK293T cells. Ubiquitination of TβRI was detected by IP of MYC-tagged constitutively active TβRI (caTβRI) in HA-Ubiquitin (HA-Ub)-transfected HEK293T cells with or without overexpression of CY-wt or CY-CA overexpression (**B**) or without (pLKO-EV) or with CY knockdown (**C**). All the groups were treated with MG132 (5 μM) for 6 h. **D** Immunoblotting analysis of TβRI and FLAG expression levels in HEK293T cells transfected with pRK5, FLAG tagged USP19-CY-wt or USP19-CY-CA expression plasmids after treatment with cycloheximide (CHX; 50 μg/mL) for the indicated times. Vinculin: loading control. **E** Quantification of the TβRI expression levels in HEK293T cells in the pRK5, CY-wt and CY-CA groups after treatment with CHX. The data were normalized to the *t* = 0 controls and expressed as the mean ± SD of three biological replicates. *P ≤ 0.05, based on unpaired Student’s *t* test. **F** Schematic diagram showing that USP19-CY induces TGF-β signaling by deubiquitinating and increasing TβRI stability

To examine the mechanism underlying the USP19-CY-induced promotion of TGF-β/SMAD signaling, we investigated whether USP19-CY targets TβRI, as USP19-CY stimulates TGF-β-induced SMAD2 phosphorylation. Both USP19-CY-wt and USP19-CY-CA strongly bound to TβRI when coexpressed in HEK293 cells (Fig. 5A). We then examined the effects of USP19-CY-wt and USP19-CY-CA on the ubiquitination of TβRI by overexpressing caTβRI and HA-tagged ubiquitin in HEK293T cells. USP19-CY-wt strongly mitigated the polyubiquitination of caTβRI; conversely, the caTβRI-associated USP19-CY-CA mutant remained highly polyubiquitinated (levels similar to that of caTβRI alone) (Fig. 5B). Moreover, knockdown of USP19-CY significantly increased the polyubiquitination of TβRI (Fig. 5C). The role of USP19-CY in regulating the TβRI stability was studied by examining TβRI expression levels after ectopic expression in HEK293T cells treated with the protein synthesis inhibitor cycloheximide (CHX). The protein half-life of TβRI was prolonged by USP19-CY-wt but not CY-CA (Fig. 5D, E). In contrast, depletion of USP19-CY strongly decreased the stability of endogenous TβRI in MDA-MB-231 cells (Supplementary Fig. S6C, D). These data suggest that USP19-CY is a DUB that acts on TβRI, and that it protects TβRI from polyubiquitination and subsequent degradation (Fig. 5F).

### The USP19-CY isoform is highly expressed in breast cancer tissues, and while herboxidiene promotes USP19-ER expression, it inhibits USP19-CY expression

**Fig. 6:**
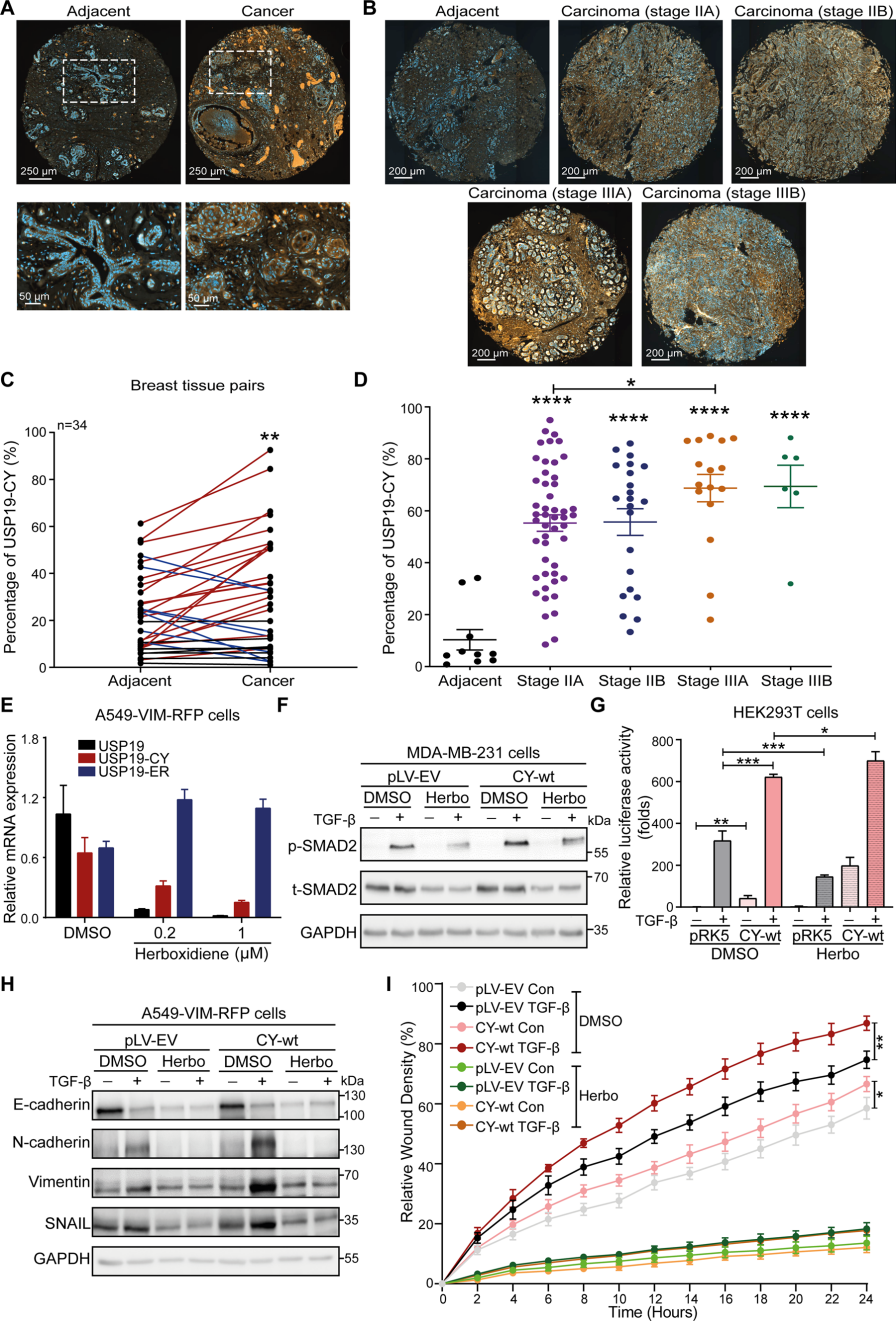
USP19-CY is highly expressed in breast cancer tissues, and USP19 mRNA splicing is regulated by herboxidiene. Representative images of USP19-CY (red) immunofluorescence staining in a human breast cancer tissue microarray containing 34 pairs of cancer adjacent tissues and cancer tissues (**A**) or cancer tissues of different stages (stage IIA, IIB, IIIA, IIIB) (**B**). Nuclei were counterstained with DAPI (blue). Large field and magnified pictures (outlined with a dotted square) are shown. Scale bar = 250 μm, 50 μm or 250 μm. **C** Quantification of the percent USP19-CY expression in pairs of breast tissues (adjacent and cancer tissues). Red lines indicate significant upregulation, and blue lines indicate downregulation of USP19-CY in cancer tissues compared to adjacent tissues; black lines indicate no significant change in USP19-CY in tissue pairs. The data are represented as the mean ± SD, tissue pairs, *n* = 34, **P < 0.01, based on a paired Student’s *t* test. **D** Quantification of percent USP19-CY expression in breast cancer adjacent tissues and cancer tissues of different stages. The data are expressed as the mean ± SD, adjacent tissues, *n* = 10; adenocarcinoma (stage IIA), *n* = 49; adenocarcinoma (stage IIB), *n* = 22; adenocarcinoma (stage IIIA), *n* = 16; adenocarcinoma (stage IIIB), *n* = 6; *P ≤ 0.05, ****P < 0.0001, based on unpaired Student’s *t* test. **E** qRT‒PCR analysis of the expression of the *USP19*, *USP19-CY* and *USP19-ER* in A549-VIM-RFP cells treated with 0.2 or 1 μM herboxidiene. The data are expressed as the mean ± SD, *n* = 3 (technical replicates). **F** MDA-MB-231 cells stably infected with pLV-EV or USP19-CY-wt were pretreated with 1 μM herboxidiene (Herbo) for 24 h and then, combined with vehicle control or TGF-β (2.5 ng/mL) for 1 h, followed by immunoblot analysis of the p-SMAD2 and t-SMAD2 expression levels. GAPDH: loading control. **G** HEK293T cells transfected with pRK5 or USP19-CY-wt were pretreated with 1 μM herboxidiene (Herbo) for 24 h and then, combined with vehicle control or TGF-β (2.5 ng/mL) overnight, followed by the analysis of CAGA_12_-luciferase transcriptional responses. The data were expressed as the mean ± SD, *n* = 3 (biological replicates). *P ≤ 0.05, **P < 0.01, ***P < 0.001, based on unpaired Student’s *t* test. **H** A549-VIM-RFP cells stably infected with pLV-EV or USP19-CY-wt were pretreated with 1 μM herboxidiene (Herbo) for 24 h and then, treated with vehicle control or TGF-β (2.5 ng/mL) for 48 h. Then, immunoblotting analysis of the expression of the epithelial marker E-cadherin and mesenchymal markers N-cadherin, vimentin and SNAIL was performed. GAPDH: loading control. **I** A549-VIM-RFP cells with pLV-EV and USP19-CY-wt plasmids were pretreated with 1 μM herboxidiene for 24 h and then, incubated with vehicle control or TGF-β (2.5 ng/mL) for the indicated times. The results of the scratch assay time course were analyzed by IncuCyte. The relative wound density (closure) is presented as the mean ± SD, *n* = 3 (biological replicates). *P ≤ 0.05, **P < 0.01, based on unpaired Student’ s *t* test

We next investigated whether USP19-CY expression can be linked to the prognosis of breast cancer patients. Therefore, we performed immunofluorescence staining (IF) for the USP19-CY protein using a USP19-CY-specific antibody. The specificity and efficiency of the antibody were validated by IF staining for USP19-CY in pRK5- and USP19-CY- transfected HEK293T cell line plugs that were embedded in paraffin (Supplementary Fig. S7A). Then, we performed IF staining for USP19-CY in two tissue microarrays: one contained 34 pairs of breast cancer tissues and adjacent phenotypically normal tissues that were derived from 34 patients, and the other included breast cancer tissues of different stages (IIA, IIB, IIIA, IIB) and 10 adjacent normal tissues (Fig. 6A, B). We observed that the USP19-CY levels were higher in breast cancer tissues than in normal adjacent tissues (Fig. 6C, D). Furthermore, more advanced breast cancer tissue stages, i.e., stage IIIA and IIIB demonstrated higher expression of USP19-CY compared than breast cancer tissue stages IIA and IIB (Fig. 6D, Supplementary Fig. S7B).

The expression of USP19-ER and USP19-CY isoforms is a result of alternative splicing [27, 46]. Thus, we aimed to identify specific small molecule splicing modulators that favor USP19-ER expression at the expense of USP19-CY expression. We therefore treated cells with eight splicing modulators (Supplementary Table S2) and analyzed USP19-ER versus CY expression. qRT‒PCR analysis revealed that T025 and herboxidiene significantly inhibited the expression levels of *USP19-CY* and *USP19*, but increased the *USP19-ER* mRNA levels (Fig. 6E, Supplementary Fig. S8). Other modulators had no clear effect on the transcript levels of *USP19*, *USP19-CY* and *USP19-ER* in HEK293T, A549, MDA-MB-231 and MCF10A-Ras cells (Supplementary Fig. S8). To examine the cytotoxicity of herboxidiene, we challenged A549-VIM-REP cells with two different concentrations of this drug and studied the effect on cell viability using the MTS assay. The data showed that both the 0.2 µM and 1 µM of herboxidiene had no significant effect on cell viabilities of A549 cells with or without USP19-CY overexpression (Supplementary Fig. S9A). Next, we investigated the effect of herboxidiene on TGF-β signaling, EMT and cell migration. We observed that herboxidiene strongly inhibited the TGF-β-induced p-SMAD2 response in MDA-MB-231 cells transfected with the empty vector (pLV-EV). This inhibition was reversed by the ectopic overexpression of USP19-CY-wt (Fig. 6F, Supplementary Fig. S9B). Conversely, T025 exerted the same inhibitory effect on TGF-β-induced SMAD2 phosphorylation in MDA-MB-231 cells stably expressing pLV-EV and UP19-CY-wt (Supplementary Fig. S9C, S9D). Consistent with this finding, the ectopic expression of USP19-CY-wt significantly rescued the CAGA-luciferase activities in HEK293T cells treated with herboxidiene but in cells treated with T025 (Fig. 6G, Supplementary Fig. S9E). Furthermore, herboxidiene strongly inhibited the TGF-β-induced expression of the epithelial marker E-cadherin and mesenchymal markers, including N-cadherin, vimentin and SNAIL, indicating the various mechanisms by which herboxidiene regulates EMT in A549 cells (Fig. 6H). Furthermore, herboxidiene completely blocked the migration of A549 cells, which also confirmed this notion (Fig. 6I). Collectively, our results suggest that USP19-CY is highly expressed in breast cancer tissues. Herboxidiene (but not T025) regulates the splicing of USP19 by favoring the USP19-CY isoform over the USP19-ER isoform at the mRNA level. Consistent with this latter finding, herboxidiene inhibits TGF-β signaling, EMT and cancer cell migration.

## Discussion

### USP19-CY and USP19-ER both interact with TβRI, but play opposing roles in TGF-β/SMAD signaling

In this study, we observed the opposing roles of two USP19 isoforms in TGF-β signaling and found that both interacted with TβRI. We showed that the USP19-CY isoform promoted TGF-β/SMAD signaling, which required DUB activity. Mechanistically, we showed that USP19-CY directly deubiquitinated and stabilized TβRI in the plasma membrane. These results are consistent with those of a previous genetic gain-of-function screen in which USP19 was identified (among many other cDNAs) to promote TGF-β-induced SMAD3/4-dependent transcriptional luciferase reporter activity [23]; we confirmed that the USP19 cDNA construct used in that study was the USP19-CY isoform. In contrast to USP19-CY, we showed that the USP19-ER isoform negatively regulated TGF-β/SMAD signaling in a DUB activity-independent manner. Furthermore, USP19-ER sequestered TβRI in the ER, thereby decreasing the TβRI levels in the plasma membrane and making the cells less responsive to TGF-β. This notion was further validated using a TβRI-KDEL fusion construct that targets TβRI to the ER. Ectopic expression of TβRI-KDEL caused a comparable level of TGF-β signaling inhibition to that of USP19-ER. Indeed, the “chaperone-like” activity of USP19-ER has been proposed previously, and this activity might allow USP19-ER to promote folding by interacting with HSP90 through its CS/p23 domain [47]. This may provide a possible mechanism by which USP19-ER affects the folding of TβRI, resulting in its retention in the ER.

### Opposing roles of USP19-ER and USP19-CY in TGF-β-induced EMT, migration and invasion

In the breast and lung cancer cells that we used in our study, USP19-CY was the major isoform that was always much more highly expressed than the USP19-ER isoform. Indeed, overexpression of TGF-β has been demonstrated in human tumor models and is seen clinically in many tumors, including breast and lung cancers [48, 49]. Thus, the high expression of the USP19-CY isoform and TGF-β may have a potential correlation and affect breast and lung tumorigenesis. In our study, the opposing roles of USP19-ER and USP19-CY in TGF-β/SMAD signaling caused USP19-CY to stimulate and USP19-ER to inhibit TGF-β-induced biological processes in breast and lung cancer, including TGF-β-induced EMT and cell migration. Importantly, we observed USP19-CY promoted the extravasation of MDA-MB-231 breast cancer cells in a zebrafish xenograft model. Interestingly, USP19-ER was shown to negatively regulate the proliferation and migration of clear cell renal cell carcinoma (ccRCC) by suppressing ERK map kinase activation [50]. In another study, overexpression of USP19-ER was found to increase breast cancer cell migration and invasion, which was dependent on its catalytic activity [51]. After the deubiquitylation of LRP6 by USP19, Wnt signaling was increased, which induced cell migration and invasion [51]. In the same study, knockdown of total USP19 inhibited MDA-MB-231 cell migration [51]; this is consistent with the finding that the depletion of USP19-CY, which is the major isoform in MDA-MB-231 cells, also inhibited cell migration. Furthermore, the authors of this study also showed that USP19 depletion decreased tumor growth and metastasis in vivo. This is consistent with the critical role of USP19-CY in TGF-β-induced extravasation and metastasis of MDA-MB-231 cells in zebrafish and mouse xenograft models.

### USP19-CY is highly expressed in breast cancer tissues

Importantly, consistent with the pro-invasive/EMT effects of the USP19-CY variant, we revealed that USP19-CY is more highly expressed in breast cancer tissues than in phenotypically normal adjacent tissues, and the higher expression level is related to more advanced cancer stages. This offers a possibility that USP19-CY expression might be linked to poor prognosis in breast cancer patients, but further survival analysis of patients with differential USP19-CY needs to be performed to validate this hypothesis. Indeed, a previous study in which high expression of USP19 was found to be associated with a significantly lower frequency of distant relapse-free survival in early breast cancer patients [51]. Additionally, elevated USP19 expression was observed in gastric cancer cells and tissues, and gastric cancer patients with high levels of USP19 expression had poor survival [52]. Although previous studies did not specify which USP19 isoform was examined, these studies can still offer some evidence of the positive roles of USP19-CY in tumorigenesis due to its predominant expression in most cancers. However, an analysis of the isoform expression signatures that are associated with tumor stages in kidney renal clear cell carcinoma (KIRC) showed that uc003cvz.3, encoding for the cytosolic isoform of USP19, was significantly decreased in patients with stage IV KIRC, whereas higher uc003cvz.3 expression suggested improved survival rates [53]. Therefore, anti-tumor or pro-tumor effects mediated by USP19-CY may differ depending on cancer subtype.

### Roles of splicing in cancer progression

Multiple studies have highlighted the frequent changes in splicing in cancer and have shown a causal role of splice variant expression in contributing to cancer progression [54, 55]. For example, CD44 variant isoforms (CD44v) that arise from the inclusion of one or more variable exons are expressed in epithelial cells, while the CD44 standard isoform (CD44s) is mainly expressed in mesenchymal cells. Thus, pharmacological manipulation of alternative splicing has been explored to evaluate its benefits for anticancer therapies. As such, a number of small molecule chemical compounds have been identified that inhibit the core spliceosome or the phosphorylation of splicing regulatory proteins [56]. Notably, we showed that herboxidiene functions as a USP19 splicing modulator by strongly decreasing the mRNA expression of USP19-CY but increasing the mRNA expression of USP19-ER, as the splicing always happens at the gene level, in breast and lung cancer cells. Another splicing modulator, namely, T025, had no effect on USP19 isoform ratios, but resulted in the downregulation of the USP19-CY isoform. The herboxidiene (but not T025)-induced inhibition of TGF-β signaling can be reversed by the overexpression USP19-CY, which confirms the opposing roles of the two USP19 isoforms on TGF-β signaling. Moreover, we found that herboxidiene can completely inhibit the basal expression of the epithelial marker E-cadherin, the TGF-β-induced expression of mesenchymal markers, including N-cadherin, vimentin and SNAIL, and the migration of lung cancer A549 cells. Inhibition of mesenchymal marker expression may mitigate single-cell migration/invasion. The low levels of E-cadherin may have a negative effect on the collective migration of these cancer cells. Notably, these strong inhibitory effects of herboxidiene on TGF-β signaling, EMT markers and cell migration indicate that besides USP19, herboxidiene has other targets. One previous study has reported that herboxidiene regulates the pre-mRNA splicing of p27, a key inhibitor of the cell cycle, leading to the accumulation of spliced p27 and inhibition of cyclin E-Cdk2 complex formation [56]. However, as an inhibitor of the core component of spliceosome, further clarification of more targets of herboxidiene with the precise mechanisms underlying herboxidiene-mediated inhibition of TGF-β-induced responses is warranted. Taken together, these results suggest that targeting alternative splicing with compounds such as herboxidiene has potential for cancer therapeutics.

In conclusion, our findings have demonstrated the distinct roles of two USP19 isoforms, namely, USP19-ER and USP19-CY, in regulating TGF-β signaling by targeting TβRI through different mechanisms. USP19-ER-mediated inhibition of TGF-β/SMAD signaling is causally linked to decreases in the TGF-β-induced EMT and migration of breast and lung cancer cells. In contrast, USP19-CY promotes TGF-β/SMAD-induced breast and lung cancer cell EMT, cell migration and extravasation in vitro and in vivo. Moreover, consistent with these findings, USP19-CY is highly expressed in breast cancer tissues. The identification of herboxidiene as a specific modulator of USP19 splicing and its concomitant inhibitory effects on TGF-β/SMAD signaling and cancer migration further validates the opposing roles of USP19-ER and USP19-CY in these processed. It will be interesting to explore the potential use of USP19-CY as a prognostic biomarker in breast cancer treatment and its potential use as a molecular target either by redirecting splicing to yield USP19-ER or inhibiting its deubiquitinating activity with selective small molecules.

## Supplementary Information

Below is the link to the electronic supplementary material.

Supplementary file1 (PDF 1923 KB)

## Data Availability

Not applicable.
