# Supplementary material for: Opposing USP19 splice variants in TGF-β signaling and TGF-β-induced epithelial–mesenchymal transition of breast cancer cells
Source: Cell Mol Life Sci. 2023 Jan 17;80(2):43. doi: 10.1007/s00018-022-04672-w (PMC9842591; doi:10.1007/s00018-022-04672-w)
Supplement: Supplementary file 1 — Supplementary file1 (PDF 1923 KB) [file 18_2022_4672_MOESM1_ESM.pdf]

Opposing USP19 splice variants in TGF-β signaling and TGF-β-induced epithelial-mesenchymal transition of breast cancer cells

Jing Zhang[1,2], Maarten van Dinther[1,2], Midory Thorikay[1,2], Babak Mousavi Gourabi[3], Boudewijn PT Kruithof[2,4], Peter ten Dijke[1,2]*

[1]Oncode Institute, Leiden University Medical Center, 2300 RC Leiden, The Netherlands.

[2]Cell Chemical Biology, Leiden University Medical Center, 2300 RC Leiden, The Netherlands.

[3]Dept. of Anatomy and Embryology, Leiden University Medical Center, 2300 RC Leiden, The Netherlands.

[4]HARTZ, Leiden University Medical Center, 2300 RC Leiden, The Netherlands.

J.Zhang.MCB@lumc.nl; M.Thorikay@lumc.nl; M.A.H.van_Dinther@lumc.nl; babak@Sepanta.nl; b.p.t.kruithof@lumc.nl; P.ten_Dijke@lumc.nl;

*Correspondence:

Peter ten Dijke, Dept. of Cell Chemical Biology, Leiden University Medical Center, 2300 RC Leiden, The Netherlands. Email: P.ten_Dijke@lumc.nl; Tel.: +31-71-526-9271; Fax: +31-71-526-8270

Supplementary Figures

Fig. S1. Schematic diagram of all the reported USP19 splice isoforms.

Fig. S2. Sequence alignment of the C-termini of USP19-ER and USP19-CY splice isoforms.

Fig. S3. USP19-CY isoform promotes TGF-β signalling.

Fig. S4. USP19-ER isoform inhibits TGF-β signalling.

Fig. S5. The opposite effect of USP19-ER and USP19-CY on the TGF-β-induced EMT.

Fig. S6. USP19-ER binds to endogenous TβRI and decreases its expression on cell surface, while USP19-CY promotes the stability of TβRI.

Fig. S7. USP19-CY is highly expressed in breast cancer tissues.

Fig. S8. Effect of various small molecule splicing modulators on mRNA expression levels of USP19, USP19-CY and USP19-ER in different cells.

Fig. S9. Herboxidiene regulates the mRNA splicing of USP19 by inhibiting USP19-CY and favouring USP19-ER isoform expression and inhibits TGF-β/SMAD signaling.

Fig. S10. The ELISA results of the rabbit blood serum with USP19-ER or USP19-CY specific antibodies.

Supplementary Tables

Table S1. Sequences of primers and plasmids.

Table S2. Structures and roles of splicing modulators.

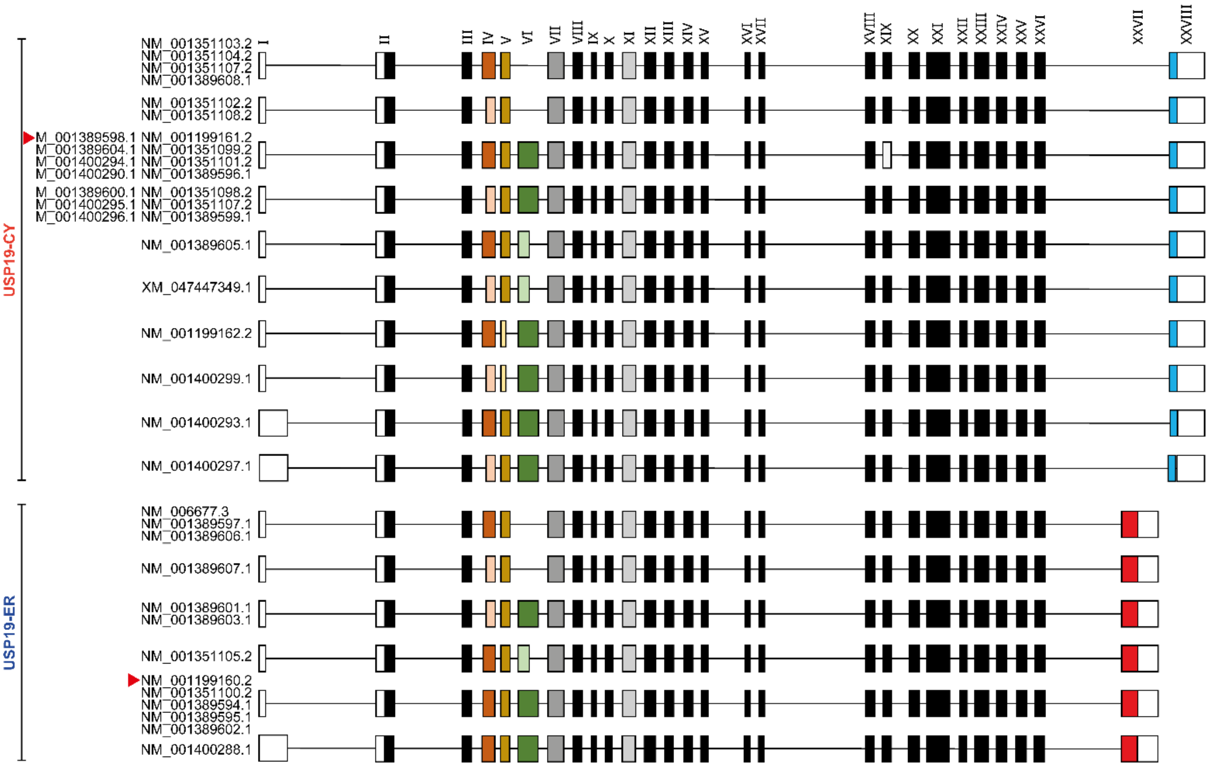

Fig. S1. Schematic diagram of all the reported USP19 splice isoforms. NCBI gene accession numbers of USP19 splice variants are indicated on the left. The mRNA encoding regions are indicated with boxes; black filled boxes correspond to coding exons that are shared among different splice variants, white boxes indicate the 5’ untranslated region (UTR) on the left or 3’ UTR on the right, the blue and red boxes correspond to the splice variants containing transmembrane region (red) or that lack that domain (blue)), respectively. Exon IV exists two variants, a short (15 amino acid deletion, indicated in light red-brown) and long variant (dark red-brown). Exon V also exists in two variants, a short (10 amino acid deletion, indicated in yellow) and a long variant (brown). Exon VI is either absent, or present in a short (50 amino acid deletion, indicated in light green) or a long variant (dark green). Exon VII (dark grey) and XI (light grey) both exist in a short or long variant (1 amino acid deletion for exon VII and a two amino acid deletion for exon XI), which are not individually indicated in the diagram. There is one transcript (NM_001400294.1) that has a 3 amino acid deletion in exon XIX (white).

The two red triangles indicate the splice variants used in this study: USP19-CY (NM_001199161.2); USP19-ER (NM_001199160.2).

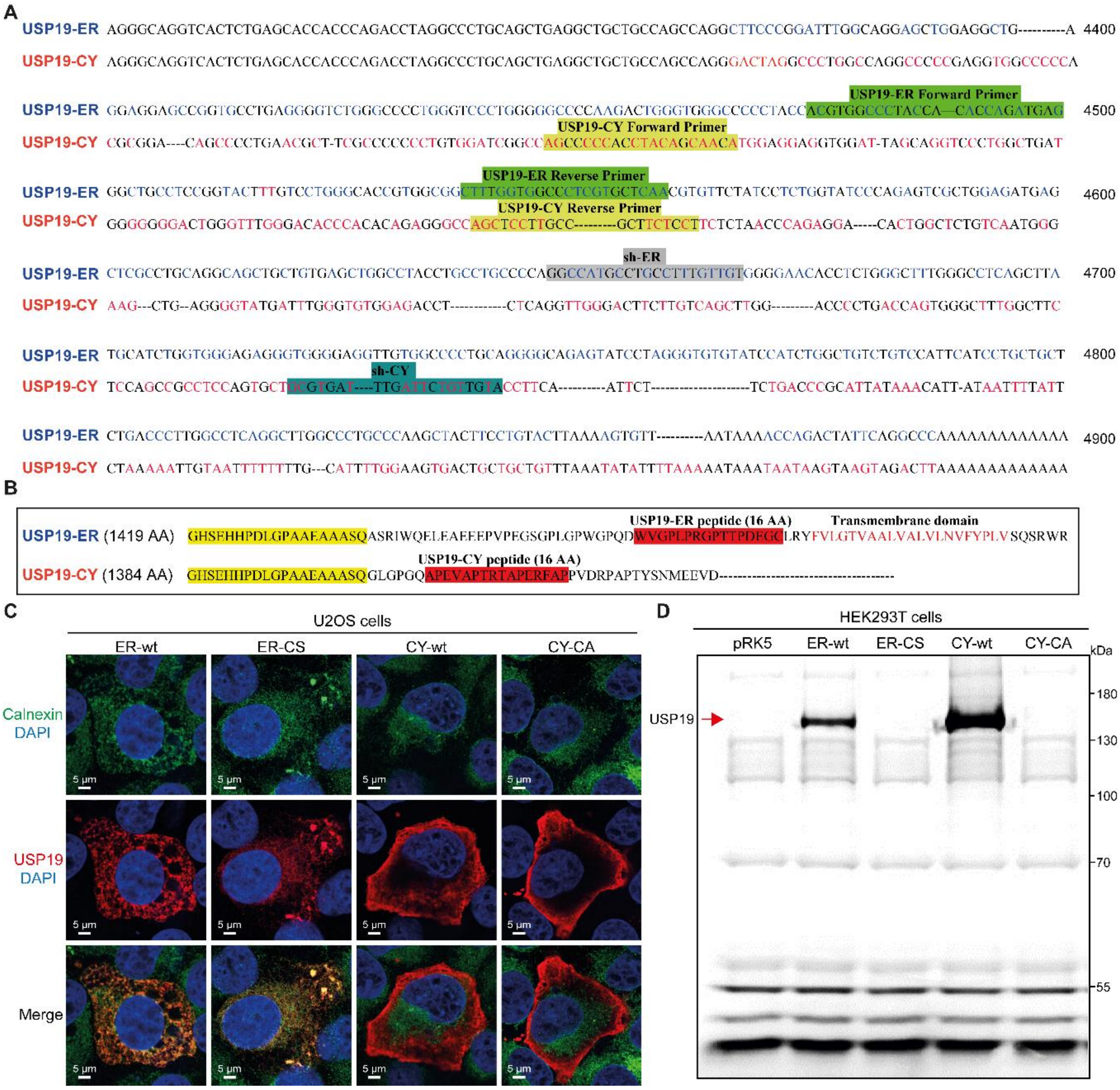

Fig. S2. Sequence alignment of the C-termini of USP19-ER and USP19-CY splice isoforms. (A) cDNA sequence alignment of parts encoding for the C-termini of USP19-ER and USP19-CY. The sequences for forward and reverse PCR primers for isoform specific mRNA expression measurement, and shRNAs for isoform specific knockdown, are highlighted. Specific nucleotides in USP19-ER cDNA sequence are shown in blue and in USP19-CY cDNA sequence are in red. **(B)** Protein sequence alignment of the C-terminal regions of USP19-ER and USP19-CY. The peptide sequences that were used to generate the antibodies for specific detection of the two isoforms are highlighted. The transmembrane domain of USP19-ER is indicated in red. **(C)** Immunofluorescence analysis of the localization of USP19 (red) and

calnexin (green) in U2OS cells transfected with FLAG-tagged USP19-CY-wt, USP19-CY-CA, USP19-ER-wt or USP19-ER-CS expression plasmids. Nuclei were counterstained with 4,6-diamidino-2-phenylindole (DAPI, blue). Images were captured with confocal microscopy. Scale bar = 5 μm. **(D)** Analysis of USP19 activities in HEK293T cells transfected with pRK5 empty vector, wild type USP19-ER (ER-wt), USP19-ER enzyme inactive mutant (ER-CS), wild type USP19-CY (CY-wt) or USP19-CY enzyme inactive mutant (CY-CA) expression plasmids using TAMRA-ubiquitin-vinyl methyl ester (VME) probe assay.

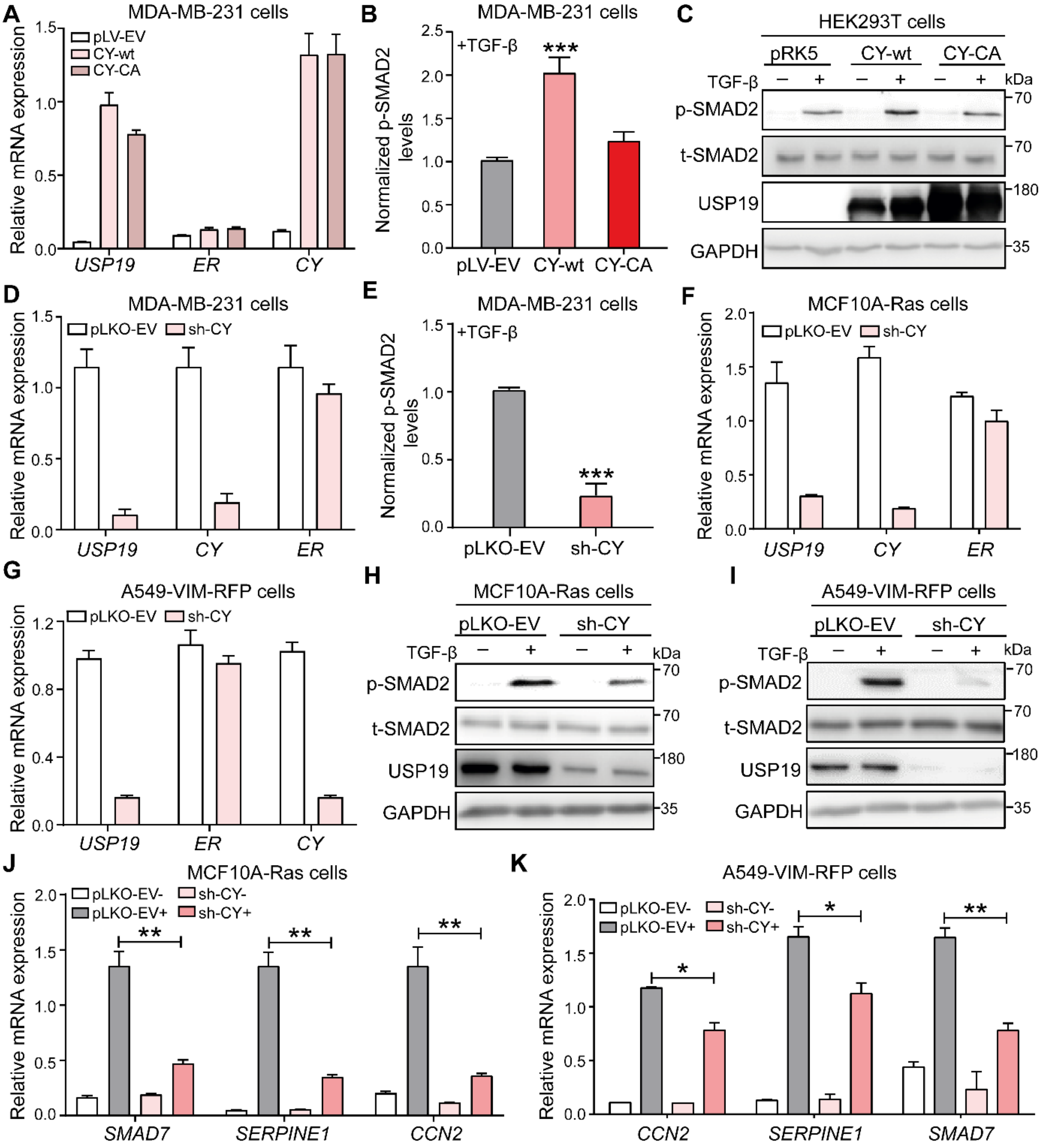

Fig. S3. USP19-CY isoform promotes TGF-β signalling. (A) qRT–PCR analysis of the *USP19*, *USP19-CY* and *USP19-ER* gene expression levels in MDA-MB-231 cells stably expressing USP19-CY-wt and USP19-CY-CA. The data are expressed as the mean ± SD, n=3 (technical replicates). **(B)** Quantification of the p-SMAD2 expression in MDA-MB-231 cells that were infected with empty vector (pRK5), USP19-CY-wt or USP19-CY-CA expression plasmids with TGF-β (2.5 ng/mL) treatment for 1 h. Results were normalized to GAPDH expression levels and expressed as the mean ± SD, n=3 (biological replicates). ***, $P < 0.001$,

based on unpaired Student's t test. **(C)** Immunoblotting analysis of the p-SMAD2, total (t)-SMAD2 and total USP19 levels in HEK293T cells that were transfected with pLV-EV, CY-wt or CY-CA plasmids after stimulation of vehicle control or TGF-β (2.5 ng/mL) for 1 h. GAPDH, loading control. **(D)** qRT–PCR analysis of the *USP19*, *USP19-CY* and *USP19-ER* mRNA expression levels in MDA-MB-231 cells without or with shRNA-mediated knock down of USP19-CY (sh-CY). The data are expressed as the mean ± SD, n=3 (technical replicates). **(E)** Quantification of the p-SMAD2 expression in USP19-CY-depleted MDA-MB-231 cells with TGF-β (2.5 ng/mL) treatment for 1 h. Results were normalized to GAPDH expression levels and expressed as the mean ± SD, n=3 (biological replicates). ***, $P < 0.001$, based on unpaired Student's t test. Expression levels of the *USP19*, *USP19-CY* and *USP19-ER* mRNA in pLKO-EV control or USP19-CY-deficient MCF10A-Ras cells **(F)** or A549-VIM-RFP cells **(G)**. The data are expressed as the mean ± SD, n=3 (technical replicates). Western blotting analysis of the p-SMAD2, t-SMAD2 and USP19 levels in MCF10A-Ras cells **(H)** or A549-VIM-RFP cells **(I)** without or with shRNA-mediated knockdown of USP19-CY (sh-CY) treated with vehicle control or TGF-β (2.5 ng/mL) for 1 h. GAPDH, loading control. qRT–PCR analysis of the TGF-β target genes, i.e., *SMAD7, CCN2* and *SERPINE1*, in USP19-CY-depleted MCF10A-Ras cells **(J)** or A549-VIM-RFP cells **(K)** in the presence of vehicle control or TGF-β (2.5 ng/mL) for 6 h. Results were normalized to *GAPDH* expression levels and expressed as the mean ± SD, n=3 (biological replicates). *, $P \leq 0.05$, **, $P < 0.01$, based on unpaired Student's t test.

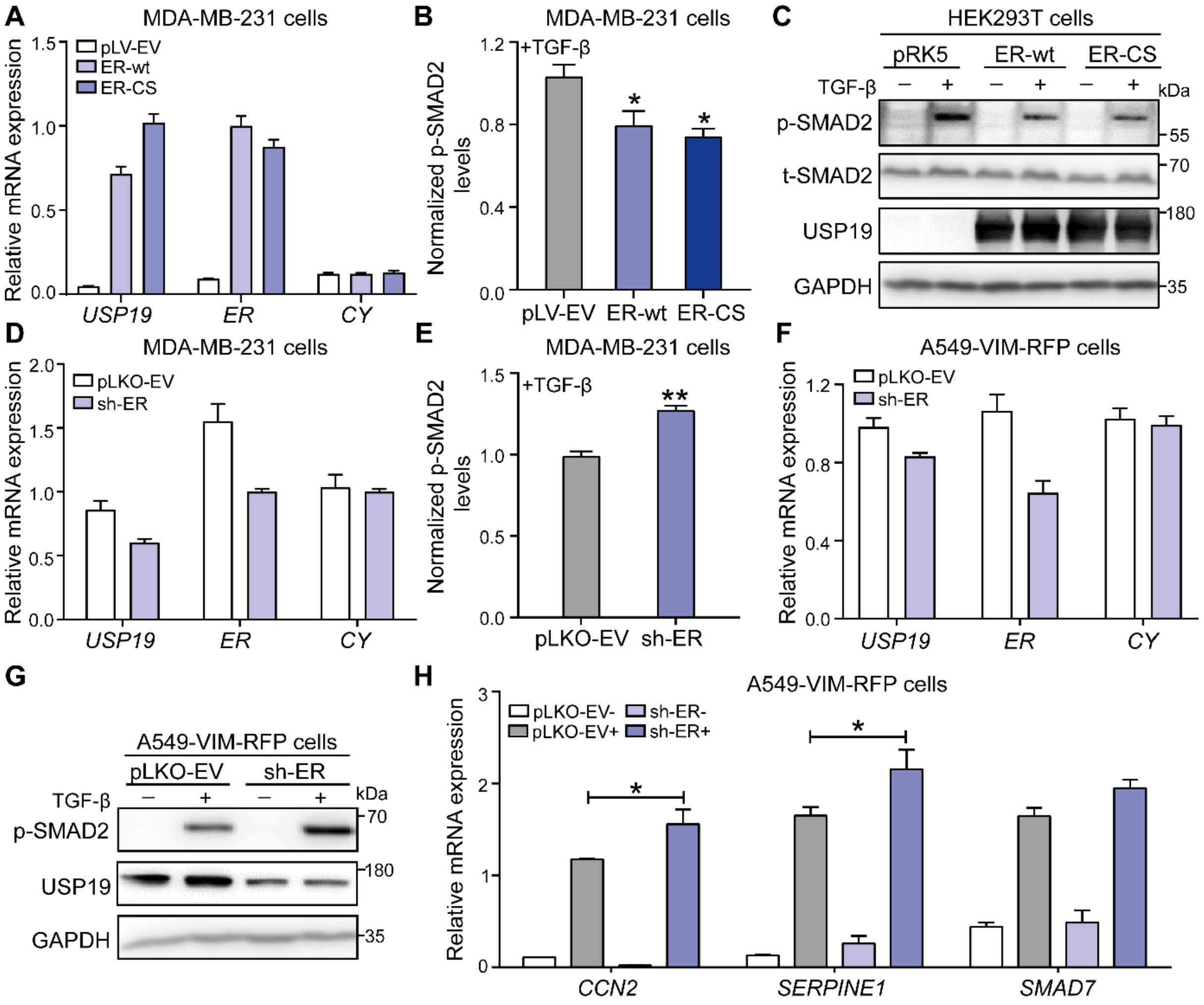

Fig. S4. USP19-ER isoform inhibits TGF-β signalling. (A) qRT–PCR analysis of the *USP19*, *USP19-CY* and *USP19-ER* mRNA expression levels in MDA-MB-231 cells stably expressed USP19-ER-wt and USP19-ER-CS. The data are expressed as the mean ± SD, n=3 (technical replicates). **(B)** Quantification of the p-SMAD2 expression in MDA-MB-231 cells that were infected with pLV-EV, ER-wt and ER-CS with TGF-β (2.5 ng/mL) treatment for 1 h. Results were normalized to GAPDH expression levels and expressed as the mean ± SD, n=3 (biological replicates). *, $P \leq 0.05$, based on unpaired Student's t test. **(C)** Western blotting analysis of the p-SMAD2, t-SMAD2 and total USP19 levels in HEK293T cells that were transfected with pRK5, ER-wt or ER-CS expression plasmids after stimulation of vehicle control or TGF-β (2.5 ng/mL) for 1 h. GAPDH, loading control. **(D)** qRT–PCR analysis of the *USP19*, *USP19-CY* and *USP19-ER* mRNA expression levels in MDA-MB-231 cells without

or with shRNA-mediated knock down of USP19-ER (sh-ER). The data are expressed as the mean ± SD, n=3 (technical replicates). **(E)** Quantification of the p-SMAD2 expression in USP19-ER-depleted MDA-MB-231 cells with TGF-β (2.5 ng/mL) treatment for 1 h. Results were normalized to GAPDH expression levels and expressed as the mean ± SD, n=3 (biological replicates). **, $P < 0.01$, based on unpaired Student's t test. **(F)** qRT–PCR analysis of the *USP19*, *USP19-CY* and *USP19-ER* mRNA expression levels in A549-VIM-RFP cells with pLKO-EV and sh-ER. The data are expressed as the mean ± SD, n=3 (technical replicates). **(G)** Immunoblotting of the p-SMAD2 and USP19 levels in A549-VIM-RFP cells without or with sh-ER treated with vehicle control or TGF-β (2.5 ng/mL) for 1 h. GAPDH, loading control. **(H)** mRNA expression levels of TGF-β target genes, i.e., *CCN2, SERPINE1* and *SMAD7* in pLKO-EV control or USP19-ER-deficient A549-VIM-RFP cells treated with vehicle control or TGF-β (2.5 ng/mL) for 6 h. Results were normalized to *GAPDH* mRNA expression levels and expressed as the mean ± SD, n=3 (biological replicates). *, $P \leq 0.05$, based on unpaired Student's t test.

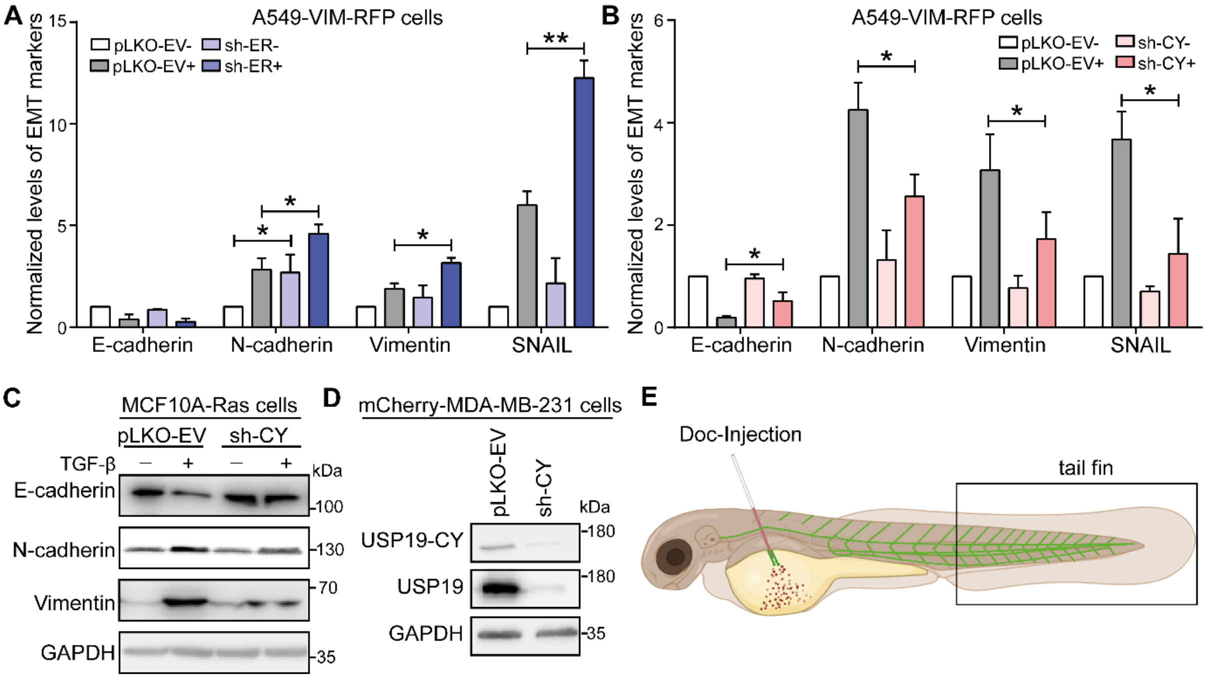

Fig. S5. The opposite effect of USP19-ER and USP19-CY on the TGF-β-induced EMT. Quantification of the EMT marker protein levels in A549-VIM-RFP cells with or without sh-ER lentivirus **(A)** or with or without sh-CY **(B)** treated with TGF-β (2.5 ng/mL) for 1 h. Results were normalized to Tubulin and GAPDH expression levels, respectively, and expressed as the mean ± SD, n=3 (biological replicates). *, $P \leq 0.05$, **, $P < 0.01$, based on unpaired Student's t test. **(C)** Western blotting analysis of the epithelial marker E-cadherin, mesenchymal markers N-cadherin and vimentin in MCF10A-Ras cells without (pLKO-EV) or with USP19-CY knockdown that were treated with vehicle control or TGF-β (2.5 ng/mL) for 2 d. GAPDH, loading control. **(D)** Immunoblotting analysis of the USP19-CY and total USP19 protein expression levels in mCherry-labelled MDA-MB-231 cells infected with pLKO-EV and sh-CY lentivirus. GAPDH, loading control. **(E)** Schematic representation of a 4-day-old zebrafish *fli*:GFP Casper embryo and the Duct of Cuvier (Doc) injection site

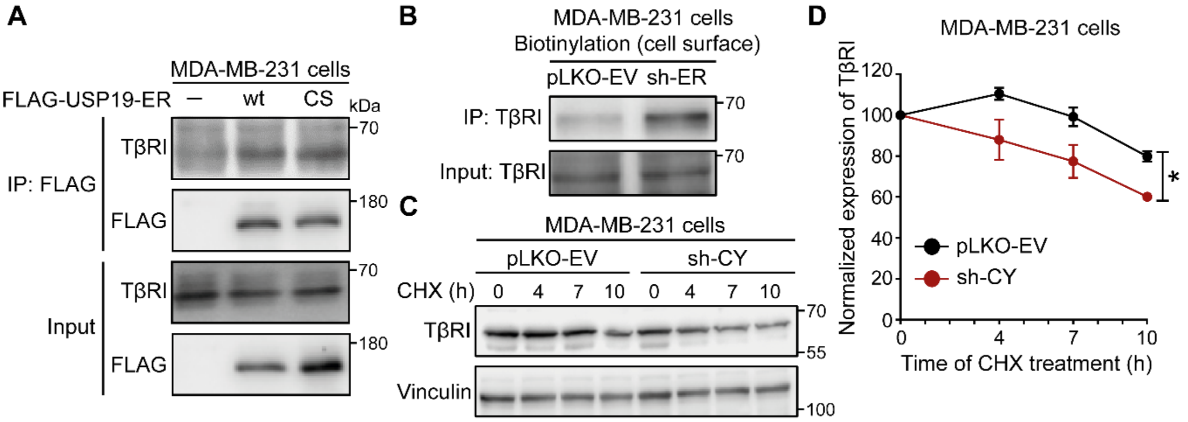

Fig. S6. USP19-ER binds to endogenous TβRI and decreases its expression on cell surface, while USP19-CY promotes the stability of TβRI. (A) Immunoprecipitation (IP) and immunoblot analysis of the interaction between USP19-ER and endogenous TβRI in MDA-MB-231 cells overexpressed with FLAG-USP19-ER-wt or FLAG-USP19-ER-CS. **(B)** MDA-MB-231 cell surface proteins without or with USP19-ER knockdown were biotinylated for 40 min at 4 °C. The biotinylated cell surface proteins were precipitated with streptavidin beads and analysed by anti-TβRI immunoblotting. **(C)** Immunoblotting analysis of the endogenous TβRI expression level in MDA-MB-231 cells infected with pLKO-EV or sh-CY after treatment with cycloheximide (CHX; 50 μg/mL) for the indicated times. Vinculin: loading control. **(D)** Quantification of the endogenous TβRI expression level in MDA-MB-231 cells in the pLKO-EV and sh-CY groups after treatment with CHX. The data were normalized to the t=0 controls and expressed as the mean ± SD of two biological independent experiments. *, $P \leq 0.05$, based on two-way ANOVA.

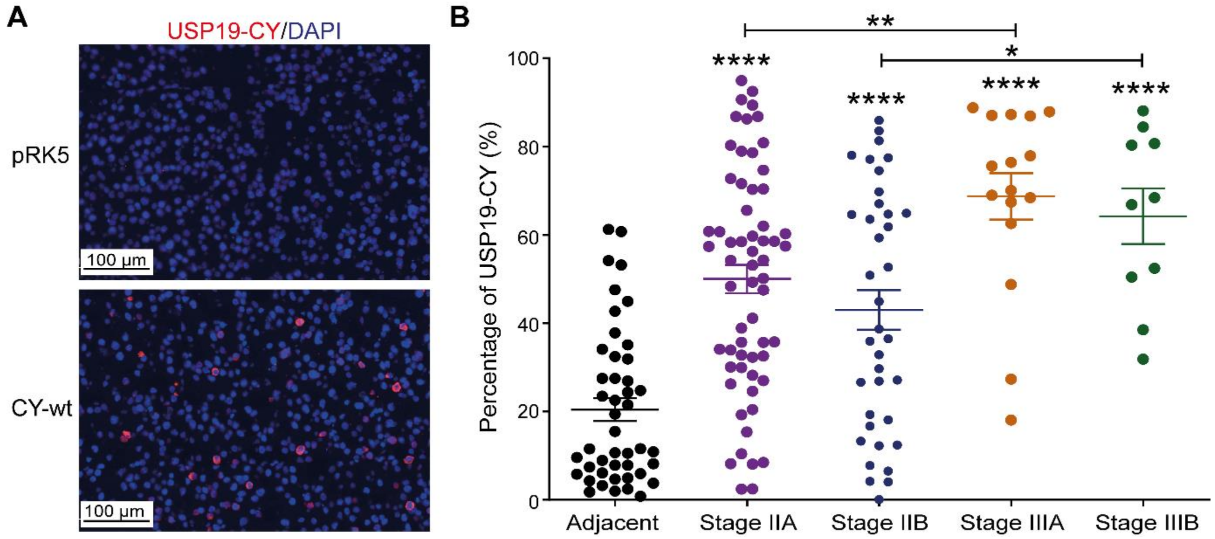

Fig. S7. USP19-CY is highly expressed in breast cancer tissues. (A) Immunofluorescence USP19-CY staining in empty vector (pRK5) or USP19-CY-wt expression plasmid transfected HEK293T cell line; plugs were formalin fixed, embedded in paraffin and sectioned. **(B)** Quantification of the precent USP19-CY expression in breast cancer adjacent tissues and different stages of cancer tissues. Adjacent tissues, n=45; adenocarcinoma (stage IIA), n=61; adenocarcinoma (stage IIB), n=37; adenocarcinoma (stage IIIA), n=16; adenocarcinoma (stage IIIB), n=10; *, $P \leq 0.05$, **, $P < 0.01$, ****, $P < 0.0001$, based on unpaired Student's t test.

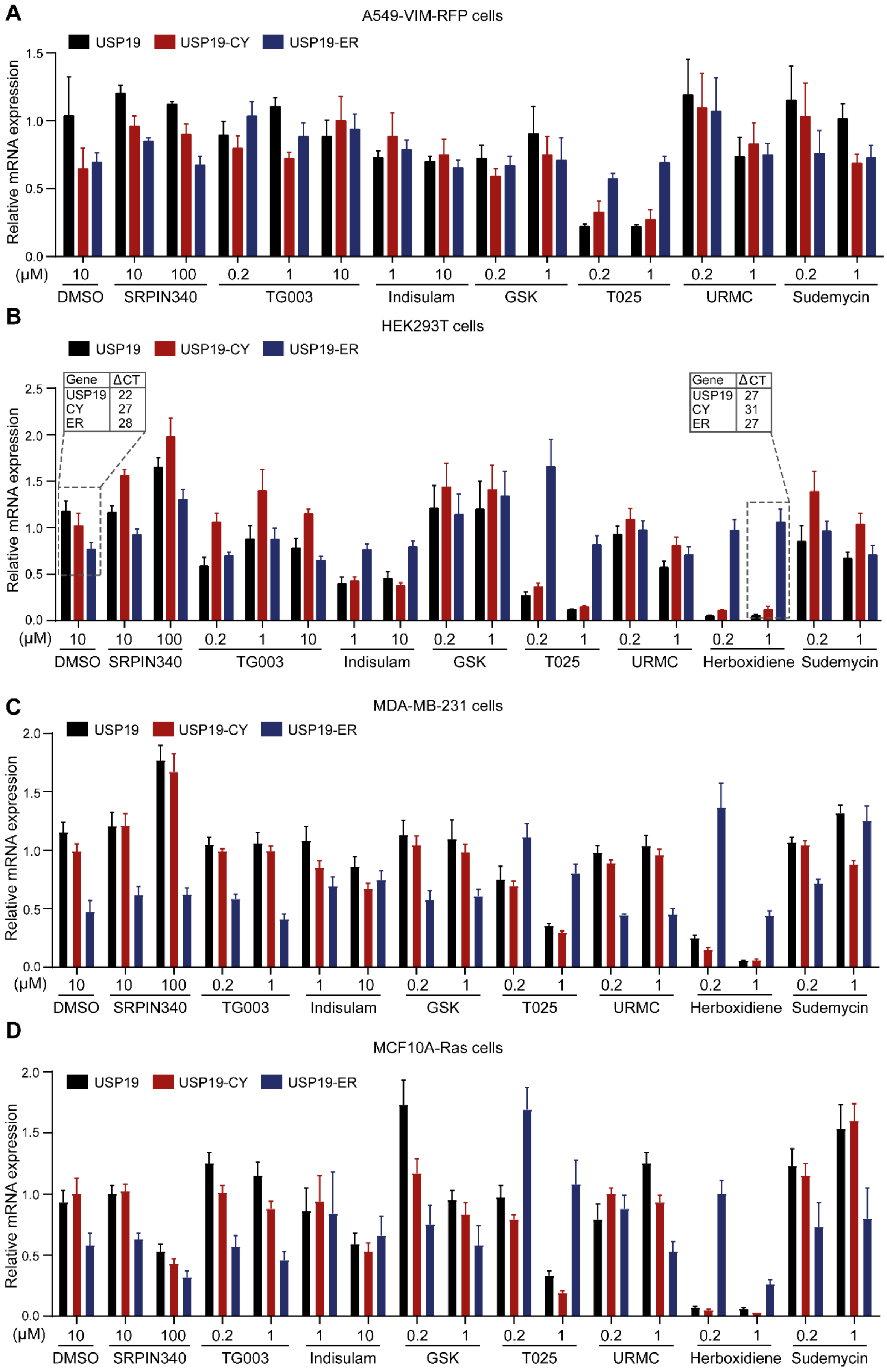
A
A549-VIM-RFP cells
USP19 USP19-CY USP19-ER
Relative mRNA expression
0.0 0.5 1.0 1.5
(μM) 10 10 100 0.2 1 10 1 10 0.2 1 0.2 1 0.2 1 0.2 1
DMSO SRPIN340 TG003 Indisulam GSK T025 URMC Sudemycin
B
HEK293T cells
USP19 USP19-CY USP19-ER
Gene ΔCT
USP19 22
CY 27
ER 28
Gene ΔCT
USP19 27
CY 31
ER 27
Relative mRNA expression
0.0 0.5 1.0 1.5 2.0 2.5
(μM) 10 10 100 0.2 1 10 1 10 0.2 1 0.2 1 0.2 1 0.2 1 0.2 1
DMSO SRPIN340 TG003 Indisulam GSK T025 URMC Herboxidiene Sudemycin
C
MDA-MB-231 cells
USP19 USP19-CY USP19-ER
Relative mRNA expression
0.0 0.5 1.0 1.5 2.0
(μM) 10 10 100 0.2 1 1 10 0.2 1 0.2 1 0.2 1 0.2 1 0.2 1
DMSO SRPIN340 TG003 Indisulam GSK T025 URMC Herboxidiene Sudemycin
D
MCF10A-Ras cells
USP19 USP19-CY USP19-ER
Relative mRNA expression
0.0 0.5 1.0 1.5 2.0
(μM) 10 10 100 0.2 1 1 10 0.2 1 0.2 1 0.2 1 0.2 1 0.2 1
DMSO SRPIN340 TG003 Indisulam GSK T025 URMC Herboxidiene Sudemycin

Fig. S8. Effect of various small molecule splicing modulators on mRNA expression levels of USP19, USP19-CY and USP19-ER in different cells. (A) A549-VIM-RFP cells **(B)** HEK293T cells **(C)** MDA-MB-231 cells **(D)** MCF10A-Ras cells. The different compound concentrations with which cells were treated for 24 h are indicated.

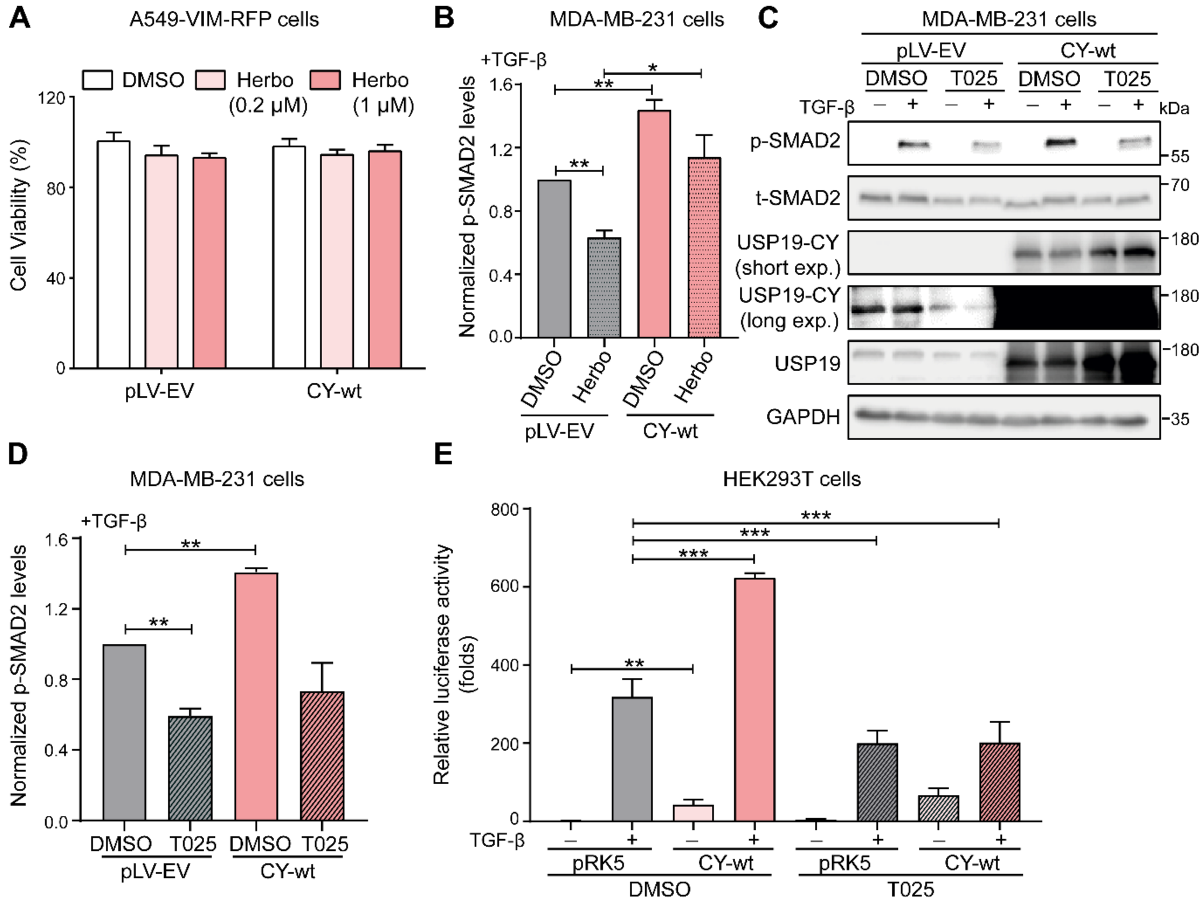

Fig. S9. Herboxidiene regulates the mRNA splicing of USP19 by inhibiting USP19-CY and favouring USP19-ER isoform expression and inhibits TGF-β/SMAD signaling. (A) The viability of A549-VIM-RFP cells with pLV-EV or USP19-CY-wt overexpression constructs was determined using MTS cell viability assay after 0.2 or 1 μM herboxidiene treatment for 24 h. **(B)** Quantification of the p-SMAD2 expression in MDA-MB-231 cells stably infected with pLV-EV and USP19-CY-wt that were pretreated with 1 μM herboxidiene (Herbo) for 24 h and then combined with TGF-β (2.5 ng/mL) for 1 h. Results were normalized to t-SMAD2 expression levels and expressed as the mean ± SD, n=3 (biological replicates). *, $P \leq 0.05$, **, $P < 0.01$, based on unpaired Student's t test. **(C)** MDA-MB-231 cells stably infected with pLV-EV or USP19-CY-wt were pre-treated with 1 μM T025 for 24 h and then combined with vehicle control or TGF-β (2.5 ng/mL) for 1 h, followed by immunoblotting analysis of the p-SMAD2, t-SMAD2, USP19-CY with short exposure time (exp.) and long

exposure time and USP19 expression levels. GAPDH: loading control. **(D)** Quantification of the p-SMAD2 expression in MDA-MB-231 cells stably infected with pLV-EV and USP19-CY-wt that were pre-treated with 1 μM T025 for 24 h and then combined with TGF-β (2.5 ng/mL) for 1 h. Results were normalized to t-SMAD2 expression levels and expressed as the mean ± SD, n=3 (biological replicates). **, $P < 0.01$, based on unpaired Student's t test. **(E)** HEK293T cells transfected with pRK5 or USP19-CY-wt were pre-treated with 1 μM T025 for 24 h and then combined with vehicle control or TGF-β (2.5 ng/mL) overnight, followed by the analysis of $CAGA_{12}$-luciferase transcriptional responses. The data were expressed as the mean ± SD, n=3 (biological replicates). **, $P < 0.01$, ***, $P < 0.001$, based on unpaired Student's t test.

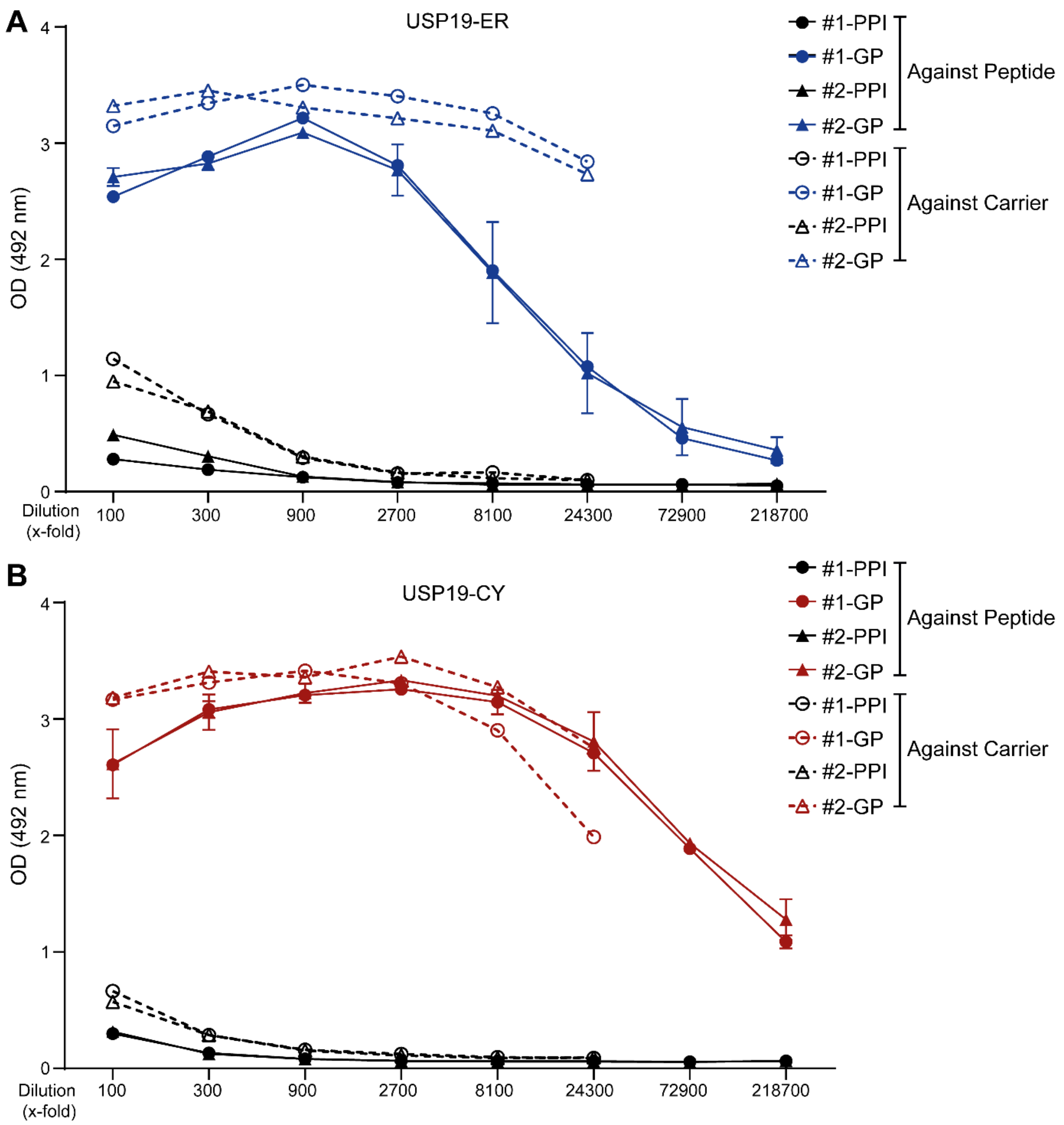

Fig. S10. The ELISA results of the rabbit blood serum with USP19-ER or USP19-CY specific antibodies. The optical density (OD) at 492 nm in the preimmune serum (PPI) or large bleed (GP) with **(A)** USP19-ER or **(B)** USP19-CY of the rabbit #1 and rabbit #2.

Table S1. Sequences of primers and plasmids.

**qRT-PCR Primers**			
**Species**	**Gene name**	**Forward (5' to 3')**	**Reverse (5' to 3')**
Human	*GAPDH*	TGCACCACCAACTGCTTAGC	GGCATGGACTGTGGTCATGAG
Human	*SERPINE1*	CACAAATCAGACGGCAGCACT	CATCGGGCGTGGTGAACTC
Human	*SMAD7*	TCCAGATGCTGTGCCTTCC	GTCCGAATTGAGCTGTCCG
Human	*CCN2*	TTGCGAAGCTGACCTGGAAGAGAA	AGCTCGGTATGTCTTCATGCTGGT
Human	*CDH1*	CAGCCGCTTTCAGATTTTCAT	CCCGGTATCTTCCCCGC
Human	*CDH2*	CAGACCGACCCAAACAGCAAC	GCAGCAACAGTAAGGACAAACATC
Human	*SNAI1*	ACCACTATGCCGCGCTCTT	GGTCGTAGGGCTGCTGGAA
Human	*USP19*	TCCGGGACTTCTTCCATGAC	GACGCCCACCAGTCCCTAGT
Human	*USP19-ER*	ACGTGGCCCTACCACACCAGATGAG	CTTTGGTGGCCCTCGTGCTCAA
Human	*USP19-CY*	AGCCCCCACCTACAGCAACA	AGCTCCTTGCCGCTTCTCCT
**PCR primers**			
**Species**	**Name**	**Forward (5' to 3')**	**Reverse (5' to 3')**
Human	*USP19-CY-CA*	CAATTTAGGCAACACCGCCTTCATGAACAGCGTC	GACGCTGTTCATGAAGGCGGTGTTGCCTAAATTG
**shRNAs**		**Target sequences (5' to 3')**	
Human	*USP19-ER*	GGCCATGCCTGCCTTTGTTGT	
Human	*USP19-CY*	GCGTGATTTGATTCTGTTGTA	

Table S2. Structures and functions of splicing modulators.

Name		Structure	Target/Mechanism of Action
SRPIN340		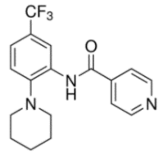	SRPIN340 inhibits the phosphorylation of the serine-arginine protein kinase (SRPK) to interfere splicing [1]
TG003		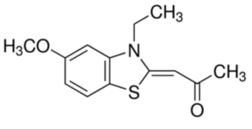	TG003 inhibits the phosphorylation of serine-arginine (SR) protein CLK kinase 1,2,4 [2]
Indisulam		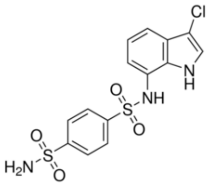	Indisulam inhibits the G1/S transition and recruits the splicing factor RBM39 to the E3 ligase substrate receptor DCAF15, resulting in altered RNA splicing and cell death [3,4]
GSK3326595		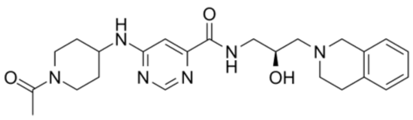	An inhibitor of protein arginine methyltransferase 5 (PRMT5), which mediates methylation of the spliceosome is a key event in spliceosome assembly [5,6]
T025		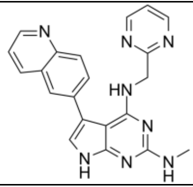	An inhibitor of Cdc2-like kinases (CLKs) that facilitate exon recognition in the splicing machinery [7]
URMC-099		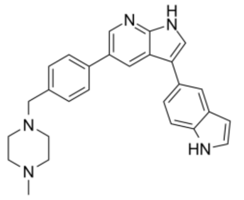	A mixed lineage kinase (MLK) inhibitor to suppress cell proliferation and migration [8]
Herboxidiene		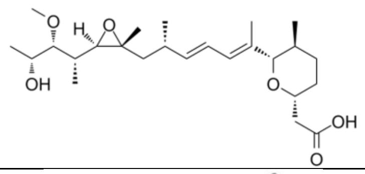	Herboxidiene noncovalently binds SF3B1, a core component of spliceosome, and alters the confirmation of SF3B1 to disrupt splicing [9]
Sudemycin D6		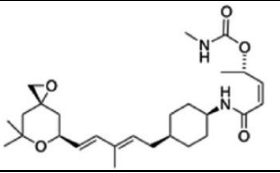	An inhibitor that targets the U2 snRNP component SF3B, and modulates alternative splicing [10]

References

1. Zhou Z, Fu XD (2013) Regulation of splicing by SR proteins and SR protein-specific kinases. Chromosoma 122 (3):191-207. doi:10.1007/s00412-013-0407-z

2. Muraki M, Ohkawara B, Hosoya T, Onogi H, Koizumi J, Koizumi T, Sumi K, Yomoda J, Murray MV, Kimura H, Furuichi K, Shibuya H, Krainer AR, Suzuki M, Hagiwara M (2004) Manipulation of alternative splicing by a newly developed inhibitor of Clks. J Biol Chem 279 (23):24246-24254. doi:10.1074/jbc.M314298200

3. Ting TC, Goralski M, Klein K, Wang B, Kim J, Xie Y, Nijhawan D (2019) Aryl Sulfonamides Degrade RBM39 and RBM23 by Recruitment to CRL4-DCAF15. Cell Rep 29 (6):1499-1510 e1496. doi:10.1016/j.celrep.2019.09.079

4. van Kesteren C, Zandvliet AS, Karlsson MO, Mathot RA, Punt CJ, Armand JP, Raymond E, Huitema AD, Dittrich C, Dumez H, Roche HH, Droz JP, Ravic M, Yule SM, Wanders J, Beijnen JH, Fumoleau P, Schellens JH (2005) Semi-physiological model describing the hematological toxicity of the anti-cancer agent indisulam. Invest New Drugs 23 (3):225-234. doi:10.1007/s10637-005-6730-3

5. Bezzi M, Teo SX, Muller J, Mok WC, Sahu SK, Vardy LA, Bonday ZQ, Guccione E (2013) Regulation of constitutive and alternative splicing by PRMT5 reveals a role for Mdm4 pre-mRNA in sensing defects in the spliceosomal machinery. Genes Dev 27 (17):1903-1916. doi:10.1101/gad.219899.113

6. Gerhart SV, Kellner WA, Thompson C, Pappalardi MB, Zhang XP, Montes de Oca R, Penebre E, Duncan K, Boriack-Sjodin A, Le B, Majer C, McCabe MT, Carpenter C, Johnson N, Kruger RG, Barbash O (2018) Activation of the p53-MDM4 regulatory axis defines the anti-tumour response to PRMT5 inhibition through its role in regulating cellular splicing. Sci Rep 8 (1):9711. doi:10.1038/s41598-018-28002-y

7. Iwai K, Yaguchi M, Nishimura K, Yamamoto Y, Tamura T, Nakata D, Dairiki R, Kawakita Y, Mizojiri R, Ito Y, Asano M, Maezaki H, Nakayama Y, Kaishima M, Hayashi K, Teratani M, Miyakawa S, Iwatani M, Miyamoto M, Klein MG, Lane W, Snell G, Tjhen R, He X, Pulukuri S, Nomura T (2018) Anti-tumor efficacy of a novel CLK inhibitor via targeting RNA splicing and MYC-dependent vulnerability. EMBO Mol Med 10 (6). doi:10.15252/emmm.201708289

8. Rhoo KH, Granger M, Sur J, Feng C, Gelbard HA, Dewhurst S, Polesskaya O (2014) Pharmacologic inhibition of MLK3 kinase activity blocks the in vitro migratory capacity of breast cancer cells but has no effect on breast cancer brain metastasis in a mouse xenograft model. PLoS One 9 (9):e108487. doi:10.1371/journal.pone.0108487

9. Hasegawa M, Miura T, Kuzuya K, Inoue A, Won Ki S, Horinouchi S, Yoshida T, Kunoh T, Koseki K, Mino K, Sasaki R, Yoshida M, Mizukami T (2011) Identification of SAP155 as the target of GEX1A (Herboxidiene), an antitumor natural product. ACS Chem Biol 6 (3):229-233. doi:10.1021/cb100248e

10. Fan L, Lagisetti C, Edwards CC, Webb TR, Potter PM (2011) Sudemycins, novel small molecule analogues of FR901464, induce alternative gene splicing. ACS Chem Biol 6 (6):582-589. doi:10.1021/cb100356k
